# MitoRibo-Tag Mice Provide a Tool for *In Vivo* Studies of Mitoribosome Composition

**DOI:** 10.1016/j.celrep.2019.09.080

**Published:** 2019-11-05

**Authors:** Jakob D. Busch, Miriam Cipullo, Ilian Atanassov, Ana Bratic, Eduardo Silva Ramos, Thomas Schöndorf, Xinping Li, Sarah F. Pearce, Dusanka Milenkovic, Joanna Rorbach, Nils-Göran Larsson

**Affiliations:** 1 Department of Mitochondrial Biology, Max-Planck-Institute for Biology of Ageing, Joseph-Stelzmann-Str. 9b, 50931 Cologne, Germany; 2 Faculty of Mathematics and Natural Sciences, University of Cologne, Albertus-Magnus-Platz, 50923 Cologne, Germany; 3 Department of Medical Biochemistry and Biophysics, Research Division of Molecular Metabolism, Karolinska Institutet, Solnavägen 9, 171 65 Solna, Sweden; 4 Max-Planck-Institute for Biology of Ageing - Karolinska Institutet Laboratory, Karolinska Institutet, Stockholm, Sweden; 5 Proteomics Core Facility, Max-Planck-Institute for Biology of Ageing, Joseph-Stelzmann-Str. 9b, 50931 Cologne, Germany

**Keywords:** mitochondria, mitochondrial biogenesis, mitochondrial DNA, mitochondrial gene expression, ribosome, translation, mitochondrial ribosome, *in vivo* mouse model, MitoRibo-Tag mice, OXPHOS

## Abstract

Mitochondria harbor specialized ribosomes (mitoribosomes) necessary for the synthesis of key membrane proteins of the oxidative phosphorylation (OXPHOS) machinery located in the mitochondrial inner membrane. To date, no animal model exists to study mitoribosome composition and mitochondrial translation coordination in mammals *in vivo*. Here, we create MitoRibo-Tag mice as a tool enabling affinity purification and proteomics analyses of mitoribosomes and their interactome in different tissues. We also define the composition of an assembly intermediate formed in the absence of MTERF4, necessary for a late step in mitoribosomal biogenesis. We identify the orphan protein PUSL1, which interacts with a large subunit assembly intermediate, and demonstrate that it is an inner-membrane-associated mitochondrial matrix protein required for efficient mitochondrial translation. This work establishes MitoRibo-Tag mice as a powerful tool to study mitoribosomes *in vivo*, enabling future studies on the mitoribosome interactome under different physiological states, as well as in disease and aging.

## Highlights

• MitoRibo-Tag mice with a tag on mL62 were generated to study mitoribosomes *in vivo*
• The mitoribosome interactome of different mouse tissues was defined with proteomics
• PUSL1 was identified as a mitoribosome-interacting protein using MitoRibo-Tag mice
• MitoRibo-Tag mice allow mitoribosome analysis under different conditions and setups

## Graphical Abstract

**Figure:**
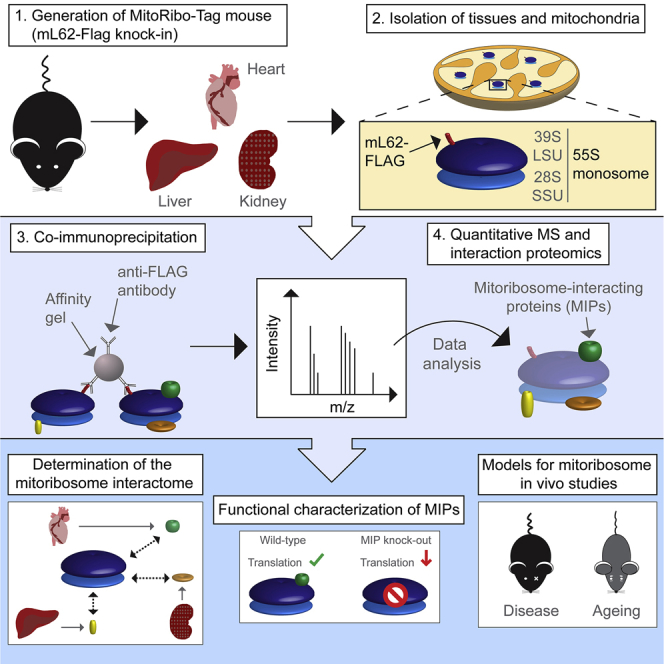

## Teaser

Busch et al. generated MitoRibo-Tag mice to study mitoribosome composition *in vivo* in different tissues. Proteomics of MitoRibo-Tag mice with defective assembly identified PUSL1 as a mitoribosome-interacting protein required for efficient mitochondrial translation. MitoRibo-Tag mice provide a tool to study mitoribosomes under different physiological conditions and in disease and aging.

## Introduction

Mitochondria are semi-autonomous eukaryotic cell organelles with important roles in key cellular processes, for example, iron-sulfur cluster biosynthesis and oxidative phosphorylation (OXPHOS; Westermann, 2010). Mammalian mtDNA is a compact ∼16.6 kb circular genome that encodes two rRNAs, 22 tRNAs, and 11 mRNAs necessary for the production of 13 essential OXPHOS proteins (Hällberg and Larsson, 2014). To synthesize mtDNA-encoded proteins, mitochondria harbor specialized ribosomes (mitoribosomes) that are 55S ribonucleoprotein complexes formed by two distinct subunits. The 28S small subunit (SSU) consists of 30 nuclear-encoded proteins and the 12S rRNA, whereas the 39S large subunit (LSU) is composed of 52 proteins, the 16S rRNA, and an integrated tRNA (Amunts et al., 2015, Greber et al., 2015, O’Brien and Kalf, 1967a, O’Brien and Kalf, 1967b). During evolution, mitoribosomes have acquired 36 organelle-specific proteins not found in bacterial ribosomes (Greber and Ban, 2016). Ribosome assembly has mostly been studied in bacteria and is a complex process occurring through an alternating series of rRNA conformation changes involving sequential binding of >200 proteins (Davis and Williamson, 2017). Mitoribosome assembly occurs co- and post-transcriptionally and depends on rRNA processing and modification and the import of nuclear-encoded proteins from the cytosol (Bogenhagen et al., 2014, Hällberg and Larsson, 2014, Rackham et al., 2016). Mitoribosome assembly and function have mostly been studied in yeast and human cultured cells, while only a few studies have been done in animal models to investigate these processes in differentiated tissues (Cámara et al., 2011, Metodiev et al., 2009, Metodiev et al., 2014, Richman et al., 2015, Wredenberg et al., 2013). The fact that cultured cells are highly proliferative and display fast protein turnover underlines the need for animal models to obtain insights into regulatory mechanisms of mitochondrial translation in tissues (Bogenhagen et al., 2018, Ruzzenente et al., 2012). Moreover, as mutations in rRNAs and mitoribosomal proteins can cause severe tissue-specific human diseases, animal models will be crucial to understand the molecular consequences of disturbed mitochondrial translation (Hällberg and Larsson, 2014, Jackson et al., 2019). We therefore generated MitoRibo-Tag mice as a versatile tool to study mitoribosome composition and the mitoribosome-interactome in different mouse tissues *in vivo*. In addition to identifying well-known translation-associated proteins, such as OXA1L, our proteomic analyses reveal a complex network of orphan and known mitochondrial protein interactions with the mitoribosome, called mitoribosome-interacting proteins (MIPs). To investigate mitoribosome biogenesis intermediates and to assess composition changes under defective assembly, we generated MitoRibo-Tag mice lacking the assembly factor MTERF4. Removal of *Mterf4* causes the loss of 55S monosomes and the accumulation of SSU and LSU mitoribosome proteins (Cámara et al., 2011). We applied quantitative proteomics in MitoRibo-Tag mice lacking MTERF4 and report that the GTP-binding protein 10 (GTPBP10) and the orphan pseudouridine synthase-like 1 (PUSL1) remain bound to the formed LSU intermediate (Lavdovskaia et al., 2018, Maiti et al., 2018, Zaganelli et al., 2017). MitoRibo-Tag mice are thus powerful tools for *in vivo* studies of mitoribosome composition during diverse physiological states, disease, and aging.

## Results

### MitoRibo-Tag Mice Stably Express mL62-FLAG

**Figure 1:**
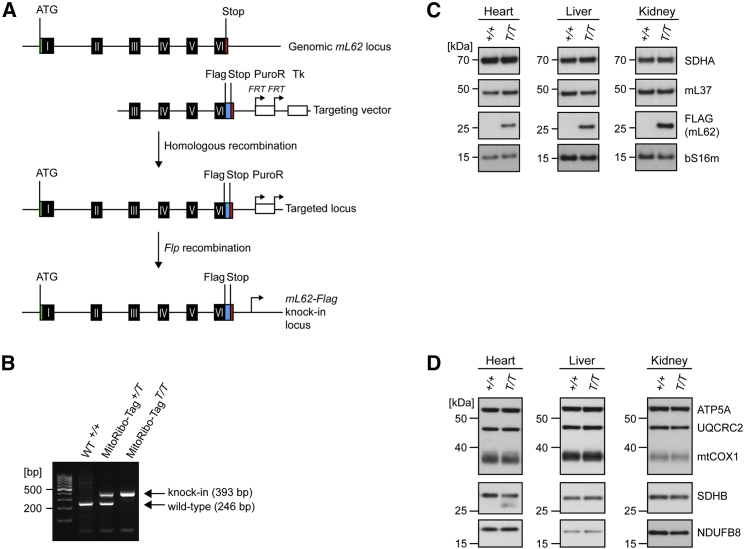
Generation of MitoRibo-Tag Mice to Study Mitoribosomes *In Vivo* (A) Targeting strategy used to generate knockin MitoRibo-Tag mice. (B) PCR analysis of WT (*+/+*), heterozygous (*+/T*), and homozygous (*T/T*) MitoRibo-Tag mice. (C and D) Western blot of 28S SSU, 39S LSU (C), and OXPHOS (D) proteins in heart, liver, and kidney mitochondria in MitoRibo-Tag and WT mice. Succinate dehydrogenase complex, subunit A (SDHA) and SDHB are shown as loading controls. Each panel is representative of four biological replicates per genotype and tissue.

To study the mitoribosome interactome *in vivo*, we generated MitoRibo-Tag knockin mice expressing the mitoribosomal protein mL62 with a FLAG-tag sequence at its carboxyl terminus. mL62 is a known LSU constituent, and from recent cryoelectron microscopy (cryo-EM) structures, it is clear that its carboxyl terminus is solvent exposed (Amunts et al., 2015, Greber et al., 2015, Richter et al., 2010). A targeting vector harboring a sequence identical to exons 3 to 6 of the mouse mL62 gene (i.e., *Mrpl58*, Ensembl: ENSMUSG00000018858) also encoding a carboxy-terminal FLAG-tag sequence was constructed and transfected into mouse embryonic stem cells (ESCs) (Figure 1A). Correctly targeted ESC clones were used to obtain chimeric mice transmitting the mutant *mL62* locus through the germline, resulting in heterozygous mice (Figure 1A). The FRT-flanked puromycin cassette was removed by crossing the heterozygous targeted mice to mice expressing the *Flp* recombinase to obtain heterozygous MitoRibo-Tag (*+/T*; *T* for transgenic) mice (Figure 1A). Correct targeting of the *mL62-FLAG* locus was verified by PCR (Figure 1B). Heterozygous MitoRibo-Tag mice were intercrossed to generate homozygous MitoRibo-Tag mice (*T/T*). MitoRibo-Tag mice were viable and indistinguishable from C57BL/6N wild-type (WT) animals and were born at mendelian ratios (data not shown), suggesting that mitochondrial function remains unaffected despite the expression of mL62-FLAG instead of mL62. We isolated mitochondria from WT and MitoRibo-Tag^*T/T*^ animals to assess the expression of the mL62-FLAG fusion protein by western blots (Figure 1C). FLAG-tagged mL62 is stably expressed in mitochondria isolated from various tissues of MitoRibo-Tag mice and does not affect the steady-state levels of SSU and LSU proteins (Figure 1C). To ensure that the expression of FLAG-tagged mL62 does not alter the mitoribosome composition or the steady-state levels of OXPHOS complexes, we analyzed protein steady-state levels by western blots and label-free quantitative mass spectrometry (LFQ-MS/MS; Figures 1D and S1). All of the mitochondrial proteins in MitoRibo-Tag mice are expressed at levels similar to WT levels, although we detected a slightly reduced expression of the mitochondrial carbamoyl-phosphate synthase in kidney mitochondria, which was characterized by an adjusted p value of 0.0346, close to the 5% false discovery rate (Figure S1). We therefore conclude that the expression of mL62-FLAG instead of mL62 in homozygous MitoRibo-Tag mice does not affect the steady-state levels of mitoribosome components or other mitochondrial proteins.

### MitoRibo-Tag Mice Have a WT Mitoribosome Assembly

**Figure 2:**
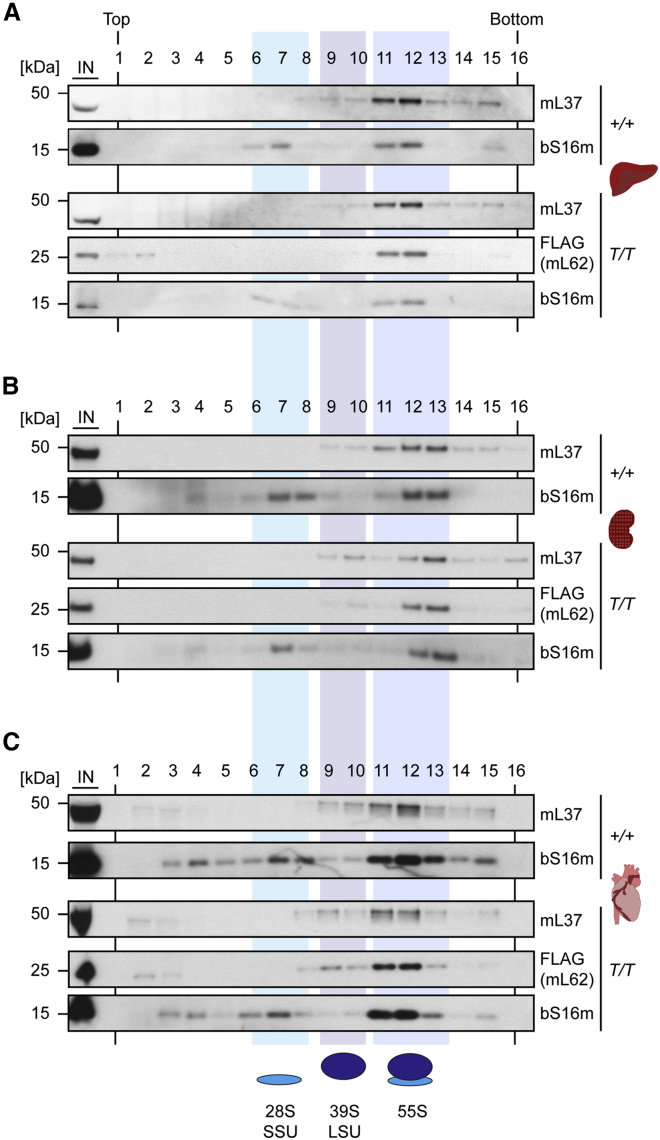
Density Gradient Analysis of MitoRibo-Tag Mitoribosomes (A–C) Density gradient analysis of MitoRibo-Tag (*T/T*) mitoribosomes from liver (A), kidney (B), and heart (C) mitochondria compared to WT (*+/+*) mitoribosomes. The lysis buffer was supplemented with digitonin, and mitochondrial lysates were loaded onto 10%–30% sucrose density gradients and centrifuged. The inputs correspond to ∼30% of the mitochondrial lysate loaded on the gradient. Fractions were taken from the top and analyzed by western blot against mL37, mL62-FLAG, and bS16m. Each panel is representative of at least three biological replicates per genotype.

To verify that mL62-FLAG incorporation in MitoRibo-Tag mice does not alter mitoribosome assembly, we analyzed mitoribosomes from heart, kidney, and liver mitochondria by sucrose-density gradient centrifugation (Matthews et al., 1982). The sedimentation profile—the assembly status of the 28S, 39S, and 55S (monosomes) mitoribosomal particles—was comparable between MitoRibo-Tag and WT mice in all of the investigated tissues (Figures 2A–2C). The majority of mL62-FLAG is stably integrated into the 55S monosome, as indicated by its co-migration with LSU protein mL37 and the SSU protein bS16m (Figure 2; Akabane et al., 2014, Richter et al., 2010). In general, most mitoribosomes seem to be assembled into 55S complexes in tissues *in vivo*, which is in contrast to most cultured cells in which 28S and 39S complexes dominate (Figures 2A–2C; Lavdovskaia et al., 2018, Maiti et al., 2018). Based on western blot and density gradient experiments, we conclude that homozygous MitoRibo-Tag mice have WT mitoribosome levels and a normal assembly state, and thus are a suitable tool to investigate mitoribosomes *in vivo*.

### MitoRibo-Tag Mice Allow Efficient Mitoribosome Purification

**Figure 3:**
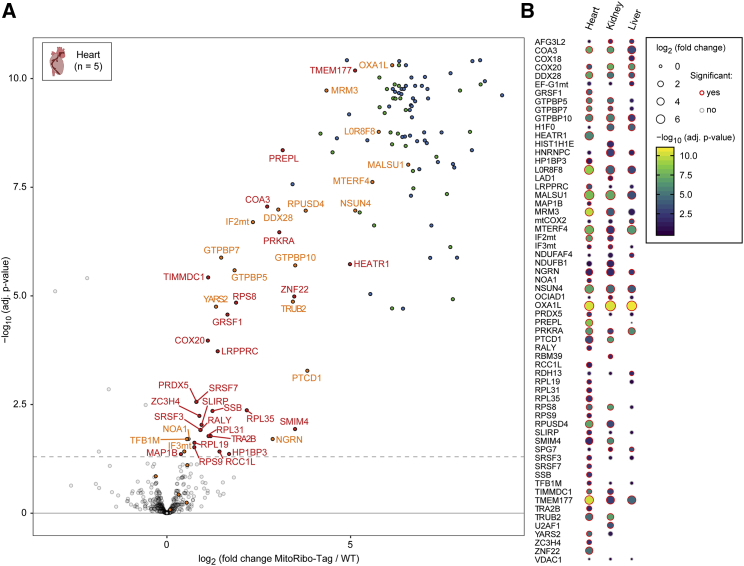
The Mitoribosome-Interactome from Heart Mitochondria of Mito-Ribo-Tag Mice (A) Mitochondria from MitoRibo-Tag and WT mice were lysed with digitonin-supplemented buffer and applied to mL62-FLAG co-immunoprecipitation followed by LFQ-MS/MS. Mitoribosomal proteins are highlighted in green (SSU) and blue (LSU). Translation-associated proteins are highlighted in orange and other significantly enriched proteins are highlighted in red. The x axis represents the fold change and the y axis indicates the adjusted p value. The dashed line represents the 5% false discovery rate. (B) Comparison of co-enriched MIPs from mitoribosome preparations from heart, liver, and kidney mitochondria identified by LFQ-MS/MS. The log_2_ (fold change; dot size), −log_10_ (adjusted p value; blue to yellow color), and significant enrichment (red circle) are indicated (5% false discovery rate). All of the results are based on five biological replicate experiments per genotype and tissue. See also Tables S1 and S2.

To investigate mitoribosome composition and associated proteins, we isolated mitoribosomes from mouse liver, kidney, and heart mitochondria with a mild lysis buffer containing digitonin, potassium (K^+^), and magnesium (Mg^2+^) to preserve subunit integrity and interactions with putative soluble or membrane-associated MIPs (Liu and Spremulli, 2000, Metodiev et al., 2009, Rackham et al., 2016, Spremulli and Kraus, 1987). We found that the elution of mitoribosomes from mouse tissues with the FLAG peptide was suboptimal for MS analysis because high levels of this peptide prevented the detection of low abundant proteins in the eluate. Instead, we eluted purified mitoribosomes by a short on-bead digestion with trypsin, obtaining high reproducibility of protein abundances (Figures 3A, 3B, and S2A; Keilhauer et al., 2015). Co-immunoprecipitation (coIP)-coupled LFQ-MS/MS of mitoribosomes from MitoRibo-Tag mice allowed the quantification of 81 of 82 (98.8%) mitoribosomal proteins to very high enrichment and confidence in liver, kidney, and heart mitochondria (Figures 3A and S2A; Suzuki et al., 2001a, Suzuki et al., 2001b). The only mitoribosomal protein that could not be quantified was bL36m, which is known to assemble very late into the mitoribosome and to be difficult to detect (Bogenhagen et al., 2018, Brown et al., 2017). Only one single peptide of bL36m was detected in two replicates from all of the coIP-experiments from mitochondria of different tissues, which allowed its identification but not quantification (Tables S1 and S2). The method specificity is supported by the absence of MRPS36 in our preparations, a previously falsely annotated mitoribosomal protein, which actually is an α-ketoglutarate dehydrogenase subunit (Heublein et al., 2014). Moreover, the coIP-LFQ-MS/MS results indicate only minor contamination using the partial on-bead trypsin elution, as the majority of proteins are annotated mitochondrial proteins (Figure 3B). Our mitoribosome coIP proteomics demonstrate that MitoRibo-Tag mice are a suitable tool to purify mitoribosomes from tissues. Moreover, this method can be used to analyze rRNA content, as mtRNAs can be detected by northern blot analyses of isolated mitoribosomes (Figure S2B).

### MitoRibo-Tag Mice Can Be Used to Determine the Mitoribosome Interactome from Differentiated Tissues

We examined the obtained coIP-LFQ-MS/MS data from MitoRibo-Tag mice to detect putative MIPs using high enrichment and confidence values as criteria (Figure 3B). Our data revealed striking tissue-specific compositional differences in the mitoribosome interactomes, with most MIPs identified in heart preparations (Figures 3A, 3B, and S3). These observations are in agreement with studies from Mootha et al. (2003) and (Pagliarini et al., 2008), who found that roughly one-third of the mitochondrial proteome is expressed tissue specifically. We verified the oxidase (cytochrome *c* oxidase) assembly 1-like (OXA1L) and mitochondrial assembly of ribosomal large subunit 1 (MALSU1) as the most co-enriched MIPs (Figure 3B; Haque et al., 2010, Rorbach et al., 2012, Wanschers et al., 2012). The fact that the mitochondrial translation initiation factors IF2_mt_ and IF3_mt_ and the mtDNA-encoded cytochrome *c* oxidase subunit II (COX2) were co-enriched indicates that our method preserves mitoribosomes in a translationally active state (Figures 3B, S3A, and S3B). Our data are consistent with reported strong interactions of mitoribosomes with the MTERF domain-containing protein 2, the NOP2/Sun RNA methyltransferase family member 4 (MTERF4:NSUN4) complex, and the recently described 16S rRNA module, composed of neugrin (NGRN), the pseudouridine synthases RNA pseudouridylate synthase domain-containing protein 4 (RPUSD4) and the tRNA pseudouridine synthase B (TruB) family member 2 (TRUB2), and the pentatricopeptide repeat-containing protein 1 (PTCD1; Figure 3B; Antonicka et al., 2017, Arroyo et al., 2016, Cámara et al., 2011, Metodiev et al., 2014, Perks et al., 2018). Several proteins involved in mitochondrial RNA metabolism co-enriched with mitoribosomes, including the mitochondrial rRNA methyltransferase MRM3, the DEAD-box RNA helicase 28 (DDX28), the G-rich RNA sequence binding factor 1 (GRSF1), the LRPPRC:SLIRP complex, and the tyrosyl-tRNA synthetase 2 (YARS2; Figure 3B; Antonicka et al., 2013, Jourdain et al., 2013, Rorbach et al., 2014, Ruzzenente et al., 2012, Tu and Barrientos, 2015). Our data corroborate recent findings that several GTP-binding proteins interact with mitoribosomes, including GTPBP5, GTPBP7, GTPBP10, and nitric oxide-associated protein 1 (NOA1; Figure 3B; Kim and Barrientos, 2018, Kolanczyk et al., 2011, Lavdovskaia et al., 2018, Maiti et al., 2018). These data support recent proposals that RNA metabolism and mitoribosome assembly are coupled and that the machineries responsible for these processes can form large assemblies, similar to the mitochondrial organization of gene expressing (MIOREX) complexes in yeast (Kehrein et al., 2015). Our data suggest the existence of previously unknown interactions of mitoribosomes with several mitochondrial inner membrane proteins involved in respiratory chain assembly, maintenance, and stability, including the complex I assembly factor TIMMDC1; the cytochrome *c* oxidase assembly factors COA3, COX18, COX20, and TMEM177; and NDUFAF4, NDUFB1, and OCIAD1 (Figure 3B; Guarani et al., 2014, Lorenzi et al., 2018, Mick et al., 2010, Shetty et al., 2018). In addition, the coIP-coupled LFQ-MS/MS experiments reveal a number of poorly characterized proteins, including prolyl endopeptidase-like protein (PREPL), retinol dehydrogenase 13 (RDH13), small integral membrane protein 4 (SMIM4), and zinc finger protein 22 (ZNF22), as previously unknown and putative MIPs, possibly involved in mitochondrial translation, that are reproducibly co-enriched with mitoribosomes (Figure 3B). To some extent, these interactions appear in a tissue-specific manner, showing the advantages of analyzing multiple tissues from MitoRibo-Tag mice (Figure 3B).

### Proteomics of MitoRibo-Tag Mice Lacking the Mitoribosome Biogenesis MTERF4 Factor Reveals Unknown MIPs

**Figure 4:**
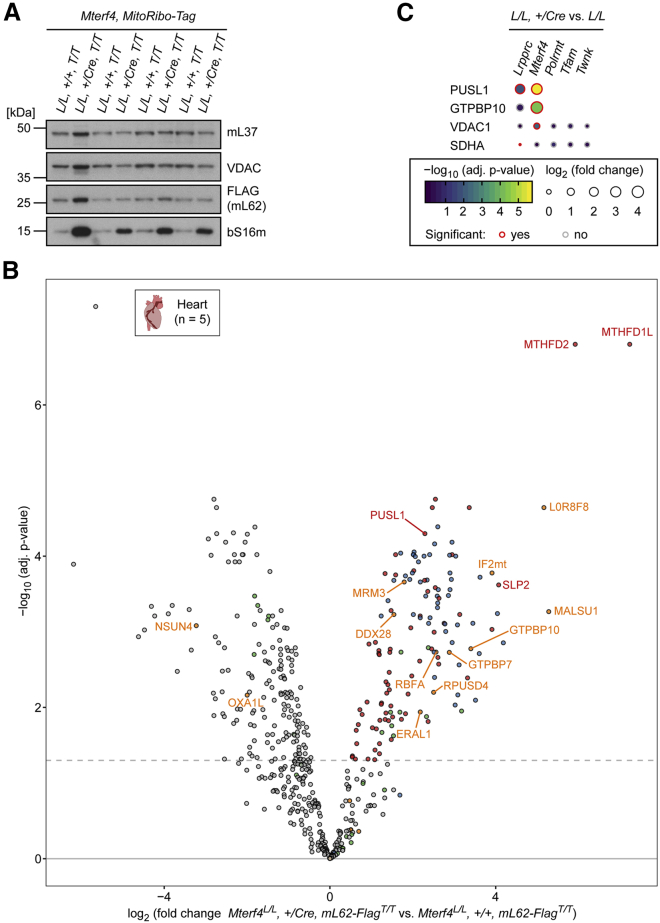
Proteomic Determination of the MTERF4 Knockout Biogenesis Intermediate (A) Western blot to detect mL37, mL62-FLAG, and bS16m in heart mitochondria of *Mterf4*^*L/L*^*, +/+, mL62*^*T/T*^ (*L/L*; control) and *Mterf4*^*L/L*^*, +/Cre, mL62*^*T/T*^ (*L/L, Cre*; knockout) MitoRibo-Tag mice. (B) mL62-FLAG-associated complexes in *L/L,* +/*Cre, T/T* versus *L/L, +/+, T/T* heart mitochondria of 18- to 20-week-old *Mterf4* MitoRibo-Tag mice. Mitochondria from *Mterf4* knockout and control mice were lysed with digitonin-supplemented buffer and applied to mL62-FLAG co-immunoprecipitation followed by LFQ-MS/MS. Mitoribosomal proteins are highlighted in green (SSU) and blue (LSU). Translation-associated proteins are highlighted in orange and other significantly enriched proteins are highlighted in red. The x axis represents the fold change and the y axis indicates the adjusted p value of *L/L,* +/*Cre, T/T* versus *L/L, +/+, T/T*. The dashed line represents a 5% false discovery rate. The results are based on five biological replicate experiments per genotype. See also Tables S1 and S2. (C) Relative fold change in the expression of GTPBP10 and PUSL1 upon the loss of *Lrpprc*, *Mterf4*, *Polrmt*, or *Twnk* in heart mitochondria of conditional knockout mice (data were adapted from Kühl et al., 2017).

To test whether MitoRibo-Tag mice can be used to detect additional MIPs involved in mitoribosomal biogenesis, we generated MitoRibo-Tag mice conditionally lacking the assembly factor MTERF4 in heart and skeletal muscle (knockout: *Mterf4*^*L/L*^*,+/Cre, mL62*^*T/T*^; control: *Mterf4*^*L/L*^*, +/+, mL62*^*T/T*^). We isolated mitochondria from these animals at age 18 to 20 weeks, corresponding to a late stage of the knockout phenotype. It has previously been shown that the MTERF4:NSUN4 complex promotes mitoribosome subunit joining (Cámara et al., 2011, Metodiev et al., 2014). *Mterf4* knockout mice cannot form 55S monosomes, resulting in increased SSU and LSU steady-state levels (Cámara et al., 2011). In agreement with our previous results, *Mterf4*^*L/L*^*, +/Cre, mL62*^*T/T*^ mice show the characteristic SSU and LSU increases compared to control animals. It should be pointed out that the mL62-FLAG fusion protein remained expressed despite the absence of MTERF4, thereby enabling purification of the subassembled 39S intermediate (Figure 4A). Next, we co-immunoprecipitated the putative 39S biogenesis intermediate accumulating in the absence of MTERF4 in *Mterf4*^*L/L*^*, +/Cre, mL62*^*T/T*^ mouse mitochondria and determined the protein composition by LFQ-MS/MS, using *Mterf4*^*L/L*^*, +/+, mL62*^*T/T*^ mitochondria as a control (Figure 4B). As expected, the majority of the significantly enriched proteins purified from *Mterf4*^*L/L*^*, +/Cre, mL62*^*T/T*^ mouse mitochondria are 39S mitoribosome constituents, whereas the putative 39S intermediate seems to lack bL32m and bL33m (Figure 4B; Table S2). The most enriched proteins were the tetrahydrofolate metabolism enzymes MTHFD1L and MTHFD2 and the mitoribosome assembly factors L0R8F8 (i.e., alternative MIEF1 protein or MIEF1 upstream open reading frame protein) and MALSU1 (Figure 4B). It is known that MTHFD1L and MTHFD2 are highly upregulated upon the loss of MTERF4, and they are therefore likely not directly binding the co-immunoprecipitated mitoribosome intermediate (Kühl et al., 2017). In addition, several mitoribosome-associated proteins, such as IF2_mt_, and assembly factors, such as DDX28, Era-like 12S rRNA chaperone 1 (ERAL1), GTPBP7, MRM3, the mitochondrial ribosome-binding factor A (RBFA), and RPUSD4, were co-enriched with the 39S intermediate from *Mterf4*^*L/L*^*, +/Cre, mL62*^*T/T*^ mitochondria (Figure 4B; Arroyo et al., 2016, Dennerlein et al., 2010, Rorbach et al., 2012, Rozanska et al., 2017, Tu and Barrientos, 2015, Wanschers et al., 2012). The SSU-associated proteins ERAL1, IF2_mt_, RBFA, and a few SSU proteins, albeit of lower adjusted p value and enrichment, could also be part of the 39S mitoribosome intermediate complex upon the loss of MTERF4, although the exact molecular nature of these interactions remains to be elucidated (Figure 4B). Moreover, among the highly significant co-enriched proteins were the recently described GTPBP10 and the orphan protein pseudouridine synthase-like 1 (PUSL1; Figure 4B; Lavdovskaia et al., 2018, Maiti et al., 2018, Zaganelli et al., 2017). GTPBP10 and PUSL1 are specifically upregulated in mouse models with defective mitoribosome assembly or poor coordination of mitochondrial translation, such as *Mterf4* and *Lrpprc* knockout mice, but not in mtRNA polymerase (*Polrmt*), mitochondrial transcription factor A (*Tfam*), or Twinkle mtDNA helicase (*Twnk*) knockout animals, with a global decrease in tRNAs and rRNAs (Figure 4C).

### GTPBP10 Enables Mitoribosome Assembly in a GTP-Dependent Manner

The identification of the recently characterized GTPBP10 as a constituent of the 39S intermediate from *Mterf4*^*L/L*^*, +/Cre, mL62*^*T/T*^ mice verifies the power of MitoRibo-Tag mice as a tool to study mitoribosome composition and assembly *in vivo*. During preparation of this manuscript, two independent studies showed that GTPBP10 is exclusively localized to mitochondria and required for biogenesis of the 39S subunit, although the exact mechanism remains unknown (Lavdovskaia et al., 2018, Maiti et al., 2018). To further examine the function of GTPBP10, which remains bound to the 39S intermediate formed in *Mterf4* knockout animals, we generated a Flp-In T-REx HEK293 cell line (HEK293T) to express GTPBP10 with a C-terminal FLAG-tag. We co-immunoprecipitated GTPBP10-associated complexes from mitochondria and assessed the interaction with the mitoribosome in the presence of the non-hydrolysable GTP analog guanosine-5′-(β,γ-imido)triphosphate (GDPNP), which has been shown to inhibit the bacterial GTPBP10 homolog ObgE, using LFQ-MS/MS and western blots (Figures S4A and S4B). We found that GDPNP strongly increased the number of 44 LSU proteins purified with GTPBP10-FLAG, verifying the recently described interaction with the 39S subunit, and additionally showing that GTP hydrolysis enables the dissociation of GTPBP10 from the mitoribosome (Figures S4A and S4B). It should be mentioned that the proteomic analysis of GTPBP10-associated complexes from mitochondrial lysates incubated with GDPNP revealed co-enrichment of a few non-ribosomal proteins (Figure S4A; Tables S1 and S2). This may result from the GDPNP treatment or GTPBP10-FLAG expression-associated side effects (Lavdovskaia et al., 2018, Maiti et al., 2018). Next, we assessed the effects of *GTPBP10* loss on cell growth and mitoribosome assembly by generating *GTPBP10* CRISPR-Cas9 knock-out HEK293T cells, confirmed by western blots (Figure S4D) and sequencing (data not shown). We obtained two *GTPBP10* knockout cell lines, both characterized by a severe growth defect in galactose medium, indicating a highly reduced OXPHOS capacity (Figure S4D). The growth phenotype of *GTPBP10* knockout cells was completely reversed by the re-expression of GTPBP10 (Figure S4D). In agreement with Maiti et al. (2018), we also observed that the genetic depletion of *GTPBP10* in cells decreased LSU (uL3, mL49, and bL28m) and slightly decreased SSU (uS15m, mS22, and uS17m) protein steady-state levels (Figure S4C). Moreover, removal of *GTPBP10* almost completely abolished *de novo* mitochondrial translation (Figure S4E). In summary, these results show that MitoRibo-Tag mice can be used to identify unknown MIPs and that GTP hydrolysis by GTPBP10 is required for mitoribosome assembly and consequently mitochondrial translation (Figure S4).

### PUSL1 Is a Mitochondrial Matrix Protein

**Figure 5:**
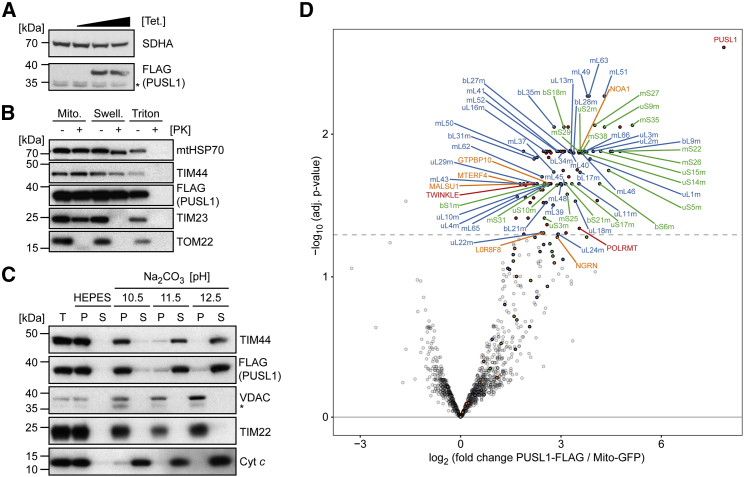
PUSL1 Is a Mitochondrial Matrix Protein and Interacts with the Mitoribosome (A) Western blot analysis of PUSL1-FLAG expression induced by tetracycline (at 0, 10, 50, or 100 ng/mL final concentration) in control and *PUSL1-FLAG* HEK293T cells. SDHA is used as a loading control. (B) Protease protection assay to assess submitochondrial localization of proteins. Mitochondria (Mito., non-treated) were swollen in hypotonic buffer (Swell.) or lysed with 1% triton X-100-supplemented buffer (triton). Samples were left untreated (−) or treated (+) with 50 μg/mL proteinase K (PK). (C) Western blot analysis of mitochondrial proteins incubated in HEPES buffer (negative control) or sodium carbonate at indicated pH values. Total (T), pellet (P), and supernatant (S) correspond to fractions obtained before and after extraction and centrifugation. (D) LFQ-MS/MS of co-immunoprecipitated PUSL1-FLAG-associated complexes using digitonin to lyse the mitochondria of HEK293T cells expressing PUSL1-FLAG (n = 4) or Mito-GFP (control, n = 3). Mitoribosomal proteins are highlighted in green (SSU) and blue (LSU). Translation-associated proteins are highlighted in orange and other significantly enriched proteins are highlighted in red. The x axis represents the fold change and the y axis indicates the adjusted p value of PUSL1-FLAG versus Mito-GFP. The dashed line represents a 5% false discovery rate. See also Tables S1 and S2.

We were intrigued by the identified interaction between the putative mitochondrial tRNA pseudouridine synthase PUSL1 and the mitoribosome. PUSL1 was co-enriched with the 39S mitoribosome intermediate formed in the absence of MTERF4 in mouse mitochondria (Figure 4B). We analyzed the predicted amino acid sequences of human (Ensembl: ENST00000379031.9) and mouse (Ensembl: ENSMUSG00000051557) PUSL1 and found that both variants harbor very similar mitochondrial targeting sequences within their first 38 amino acids (data not shown; Bateman et al., 2017, Claros and Vincens, 1996, Mitchell et al., 2019, Sievers et al., 2011). Human and mouse PUSL1 share two conserved pseudouridine synthase domains at the N- and the C termini, which are homologous to the bacterial tRNA pseudouridine synthase A (TruA) family of pseudouridine synthases. Moreover, PUSL1 has been predicted to localize to human mitochondria in several recent high-throughput studies (Calvo et al., 2016, Pagliarini et al., 2008, Rhee et al., 2013). We found that commercial PUSL1 antibodies are not working reproducibly, being accompanied by many cross-reacting bands, therefore not allowing an adequate localization of PUSL1 by biochemical fractionation. To investigate the submitochondrial localization of PUSL1 and to confirm its interaction with the mitoribosome, we generated a HEK293T cell line to inducibly express FLAG-tagged PUSL1 (Figure 5A). We performed a protease protection assay and found that PUSL1 is protected from degradation by proteinase K added to mitochondria with an intact or a disrupted outer membrane, and thus behaves as the known mitochondrial matrix proteins TIM44 and HSP70 (Figure 5B). Upon sodium carbonate extraction, PUSL1 was extracted from the membrane at high basic pH similar to the inner membrane-associated protein TIM44, whereas the integral inner membrane protein TIM22 and the integral outer membrane protein VDAC were not solubilized (Figure 5C). The intermembrane space protein cytochrome *c* (Cyt *c*) associated with the inner membrane was soluble under all different sodium carbonate extraction conditions (Figure 5C). Using immunofluorescence of transiently transfected HeLa cells, we verified that the PUSL1-FLAG fusion protein exclusively localizes to mitochondria (Figure S5A), as recently observed by Zaganelli et al. (2017). In summary, by using a combination of biochemical fractionation and immunofluorescence techniques, we demonstrate that PUSL1 is a mitochondrial matrix protein peripherally attached to the mitochondrial inner membrane (Figures 5B, 5C, and S5).

### PUSL1 Interacts with the Mitoribosome

To further examine the nature of the interaction of PUSL1 with the mitoribosome, we isolated mitochondria from HEK293T cells stably expressing PUSL1-FLAG and co-immunoprecipitated PUSL1-associated complexes to determine their composition by LFQ-MS/MS (Figure 5D). We found that PUSL1 interacts with mitoribosomal proteins, including 20 SSU and 36 LSU constituents, as well as the mitoribosome assembly factors L0R8F8, GTPBP10, MALSU1, MTERF4, and NOA1. These results show that PUSL1 is indeed an orphan MIP, with a possible role in mitochondrial translation (Figure 5D; Brown et al., 2017). MitoRibo-Tag mice, combined with conditional knockout of mitoribosome biogenesis factors, thus provide powerful tools to discover novel MIPs, which may not be observed under basal physiological conditions (Figures 3B and 4B).

### Loss of PUSL1 Decreases *De Novo* Mitochondrial Translation

**Figure 6:**
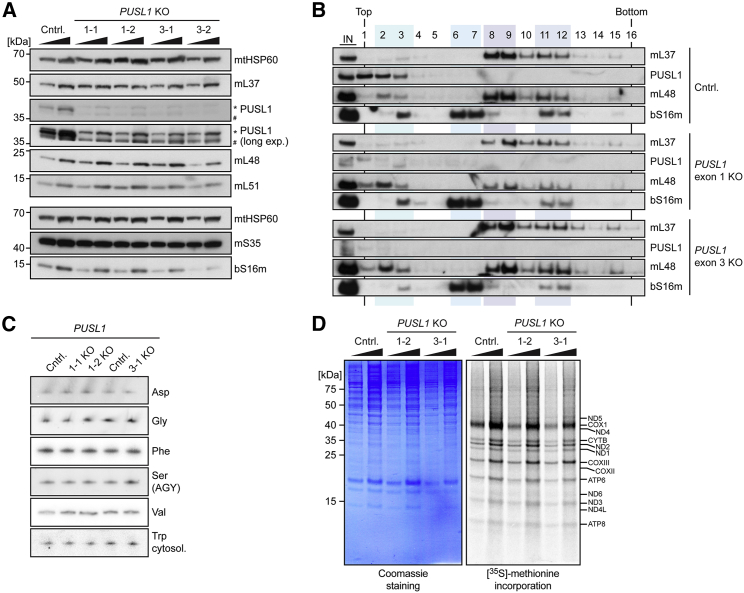
Loss of PUSL1 Affects Mitoribosome Protein Steady-State Levels and Leads to Decreased Mitochondrial *De Novo* Translation (A) Western blot analysis of mitochondrial protein steady-state levels in *PUSL1* knockout mitochondria versus mitochondria from control HEK293T (Cntrl.) cells. Crude mitochondrial protein, 10 and 20 μg, were loaded. CRISPR-Cas9 targeted exons of *PUSL1* (first digit) and respective clones (second digit) used for the experiment are indicated by 1-1, 1-2, 3-1, and 3-2. A short and a long exposure using the commercial PUSL1 antibody are shown. Asterisk and cross-hatch indicate cross-reacting bands. (B) Density gradient analysis of mitoribosomes in *PUSL1* knockout mitochondria and control mitochondria from HEK293T cells (n = 2). Mitochondrial lysates were loaded onto 10%–30% sucrose density gradients and centrifuged. The inputs correspond to ∼16% of loaded mitochondrial lysate. Fractions were taken from the top and analyzed by western blot against the indicated proteins. (C) Northern blot analysis of mtDNA-encoded tRNAs. The cytosolic tRNA tryptophan was used as a loading control. (D) Representative *in cellulo* [^35^S]-methionine pulse-labeling experiment in *PUSL1* knockout cells versus HEK293T control cells (n = 2 per genotype and cell line) of whole-cell lysates, 10 and 20 μg protein, were loaded.

To gain insight into PUSL1 function, we applied CRISPR-Cas9 genome editing with two different guide RNAs (gRNAs) targeting exon 1 and exon 3 of *PUSL1* in HEK293T cells (Table S3). Sequencing revealed that the gRNAs induced a single base insertion that led to premature stop codons and the complete loss of PUSL1 on western blots (Figure 6A). The *PUSL1* knockout cells showed a slight increase in 39S subunit constituents (e.g., mL48 on western blots) (Figure 6A). Analysis of mitoribosome assembly on sucrose gradients revealed no marked differences between *PUSL1* knockout and control mitochondria (Figure 6B). As PUSL1 is homologous to the bacterial tRNA pseudouridine synthase TruA, we also assessed the levels of mtDNA-encoded tRNAs in *PUSL1* knockout cells by northern blotting but found no differences from control cells (Figure 6C). Most importantly, *PUSL1* knockout cells had a decreased mitochondrial *de novo* translation rate (Figure 6D). These results are consistent with *PUSL1* small interfering RNA (siRNA) knockdown experiments, in which the completely abolished expression of PUSL1 in HEK293T cells was also accompanied by decreased mitochondrial translation (Figures S6A and S6B). Furthermore, proteomic analysis of mitochondria from siRNA-treated HEK293T cells revealed that the downregulation of PUSL1 caused a significant upregulation of the mitoribosome constituent mL51 and affected several translation-associated proteins such as mitochondrial ribosome recycling factor (MRRF) and many mitochondrial tRNA synthetases (Figures S6C and S6D). In summary, our data show that PUSL1 is a MIP involved in the regulation of mitochondrial translation.

## Discussion

In this study, we present the MitoRibo-Tag knockin mice as a versatile tool to investigate the *in vivo* composition and interactome of mitoribosomes in different tissues and physiological states. Since the discovery of mitoribosomes in 1967, a number of studies have contributed to our understanding of mitochondrial translation and mitoribosome composition (O’Brien and Kalf, 1967a, O’Brien and Kalf, 1967b). Many mitoribosome assembly factors modifying essential bases or putatively stabilizing conformations of mitochondrial rRNAs, including MRPP3, MRM1–MRM3, MTERF3, MTERF4, NSUN4, PTCD1, RPUSD4, and TFB1M, have recently been described (Hällberg and Larsson, 2014, Lee et al., 2018). In addition, a few MIPs are known to specifically regulate the synthesis of mtDNA-encoded proteins mtCOX1 and mtCYT*b* in yeast and mammals (Ott et al., 2016). Furthermore, auxiliary factors such as Mba1, Mrx15, and Oxa1 in yeast and OXA1L and MITRAC complexes in mammals are known to recruit mitoribosomes to sites of cytochrome *c* oxidase assembly (Mick et al., 2012, Ott et al., 2006, Richter-Dennerlein et al., 2016, Möller-Hergt et al., 2018). Despite the available mitoribosome structures, we are still lacking the essential knowledge of proteins coordinating translation with OXPHOS biogenesis and mitoribosome assembly (Hällberg and Larsson, 2014, Ott et al., 2016). As mitoribosome assembly and translation coordination in mitochondria strongly diverge from those in bacteria or the eukaryotic cytosol, it is expected that several mitochondrial-specific translation factors and MIPs have been acquired to assist in these processes (Brown et al., 2017). In part, our knowledge of ribosome function and assembly *in vivo* is limited due to the scarcity of relevant animal models.

We generated MitoRibo-Tag mice to allow the investigation of mitoribosome composition under basal conditions and in response to mitochondrial dysfunction. We used MitoRibo-Tag mice to define the mitoribosome-interactome across different tissues, revealing tissue-specific compositional differences and unknown MIPs. The different interactome compositions may partially explain the variable tissue-specific phenotypes observed in human patients suffering from mitochondrial translation-associated diseases (Hällberg and Larsson, 2014). Therefore, MitoRibo-Tag mice are an important *in vivo* tool to investigate the tissue specificity of the mitoribosome interactome and the molecular mechanisms underlying the variable outcomes of translation-associated mitochondrial diseases.

In agreement with recently published studies, we verified that GTPBPs are important regulatory mitoribosome assembly factors *in vivo*, and our experiments provide additional evidence that GTPBP10 controls LSU biogenesis in a GTP-dependent manner. GTPBP10 may allow mitoribosome assembly to proceed only if its substrate GTP can be hydrolyzed. This idea is supported by the presented coIP-LFQ-MS/MS experiments, which show that GTPBP10 remains bound to mitoribosomal proteins in the presence of the non-hydrolysable GTP analog GDPNP. Hence, one can hypothesize that GTPBP10 prevents the entry of premature LSUs into monosome assembly to avoid diminishing the translational capacity formation of malfunctioning mitoribosomes (Lavdovskaia et al., 2018, Maiti et al., 2018).

We also show that MitoRibo-Tag mice are a suitable tool to discover yet unknown interactions or orphan MIPs and mitoribosome biogenesis intermediates, which are not identified under normal physiological conditions. By coIP-coupled proteomics in MitoRibo-Tag mice, we defined the interactome of a mitoribosome biogenesis intermediate formed in mitochondria lacking MTERF4. Similar approaches in bacteria based on a combination of genetics with proteomics or cryo-EM have led to the identification of immature ribosomal particles bound to assembly factors (Davis and Williamson, 2017). In the analysis of mammalian mitoribosomes we present here, PUSL1 and GTPBP10 were found to remain bound to a 39S assembly intermediate formed in the absence of MTERF4. PUSL1 is homologous to *Escherichia coli* TruA, which is known to pseudouridylate uridines in 17 bacterial tRNAs (Hur and Stroud, 2007, Suzuki et al., 2011). The exact molecular function of each pseudouridine in the different RNA species is not understood, but it is generally accepted that pseudouridines stabilize RNA conformations (Ofengand, 2002). Recent studies have suggested a requirement of pseudouridylation for the stability of mitochondrial tRNAs and the 16S rRNA to enable translation and mitoribosome assembly (Antonicka et al., 2017, Perks et al., 2018).

Using MitoRibo-Tag mice as a tool, we identified PUSL1 as an inner membrane-associated mitochondrial matrix protein interacting with mitoribosomes. Proteomic analysis of PUSL1-FLAG-associated complexes revealed interactions between PUSL1, mitoribosomal proteins, and several assembly factors, including GTPBP10, L0R8F8, MALSU1, MTERF4, and NOA1. One could speculate that PUSL1, together with or independently of GTPBP10, safeguards a quality control checkpoint during mitoribosome assembly as supported by the finding that the removal of these proteins reduces mitochondrial *de novo* translation. This hypothesis is further strengthened by the demonstration that the mitoribosome interactomes from MTERF4 knockout MitoRibo-Tag mice and PUSL1-associated complexes from human mitochondria partially overlap with the enrichment of GTPBP10, L0R8F8, MALSU1, and, if expressed, MTERF4. Pseudouridines could represent adaptations of the mitochondrial translation system to stabilize RNA conformations or mitoribosomal biogenesis intermediates (Ofengand, 2002, Zaganelli et al., 2017). The recent finding that several pseudouridine synthases are required for mitochondrial translation is intriguing, as there is only one known pseudouridine synthase in *E. coli*, RluD, which is strictly required for bacterial growth (Arroyo et al., 2016, Del Campo et al., 2004). It is tempting to speculate that PUSL1 chaperones mitoribosome assembly or functions via modification of the structurally integrated central protuberance tRNA. It is known that mitochondrial phenylalanine and valine tRNAs, which serve as structural constituents in the central protuberance in porcine and human mitoribosome LSUs, harbor pseudouridines at base positions 37 (phenylalanine) and 24 and 29 (valine) (Suzuki and Suzuki, 2014). It is possible that PUSL1 pseudouridylates uridines (e.g., at base position 37 [tRNA phenylalanine] or 24 [tRNA valine]), contributing to mitoribosome integrity or regulation of mitochondrial translation (Suzuki et al., 2011). It will be interesting to investigate the exact molecular role of PUSL1 in the regulation of the mitochondrial transcriptome and translatome, for example, by the application of recently developed (pseudouridine) RNA sequencing methods to identify putatively modified bases, and ribosome profiling to investigate the molecular consequences on the translation of certain mRNAs or tRNA codons.

In summary, we have generated MitoRibo-Tag mice and show that they are a valuable and versatile tool to molecularly define the mitoribosome composition and interacting proteins that are necessary for regulating mitochondrial translation *in vivo*. The technological advantages of MitoRibo-Tag mice include the possibility of analyzing mitoribosomes from different tissues and analyzing the composition of assembly intermediates obtained by the disruption of genes encoding biogenesis factors. We also envision that MitoRibo-Tag mice will be important tools to study mitoribosomes and their interactomes in different tissues, when animals are subjected to varying physiological conditions (e.g., starvation, exercise, caloric restriction or high-fat diet), or in disease states (e.g., diabetes), as well as during the normally occurring aging process.

## STAR★Methods

### Key Resources Table

**Table:** 

REAGENT or RESOURCE	SOURCE	IDENTIFIER
**Antibodies**		

bL12m / MRPL12	Sigma-Aldrich	RRID: AB_1854099; Cat#HPA022853
bL28m / MRPL28	Sigma-Aldrich	RRID: AB_10601136; Cat#HPA030594
bS16m / MRPS16	Proteintech Group	RRID: AB_2180166; Cat#16735-1-AP
Cytochrome *c*	Abcam	RRID: AB_300470; Cat#ab13575
DDDDK tag (FLAG-tag)	Abcam	RRID: AB_299216; Cat#ab1257
DYKDDDDK tag (9A3) Mouse mAb	Cell Signaling	RRID: AB_10950495; Cat#8146S
GAPDH	Abcam	RRID: AB_2107448; Cat#ab8245
GTPBP10	Sigma-Aldrich	RRID: AB_1850411; Cat#HPA021076
mL37 / MRPL37	Sigma-Aldrich	RRID: AB_1854106; Cat#HPA025826
mL48 / MRPL48	Proteintech Group	RRID:AB_2282151; Cat#14677-1-AP
mL49 / MRPL49	Proteintech Group	RRID: AB_2146189; Cat#15542-1-AP
mL51 / MRPL51	Abcam	Cat#ab235828
mS22 / MRPS22	Proteintech Group	RRID: AB_2146488; Cat#10984-1-AP
mS35 / MRPS35	Proteintech Group	RRID: AB_2146521; Cat#16457-1-AP
mtHSP60	Santa Cruz	RRID: AB_631683; Cat#sc-1052
mtHSP60 (for mouse samples)	Cell Signaling	Cat#4870S
mtHSP70	Abcam	RRID: AB_881520; Cat#ab47455
OXPHOS cocktail human	Abcam	Cat#ab110411
OXPHOS cocktail mouse	Abcam	RRID: AB_2629281; Cat#ab110413
PUSL1	Sigma-Aldrich	RRID: AB_10601251; Cat#HPA032057
SDHA	Invitrogen	RRID: AB_1501830; Cat#459200
TIM22	Proteintech Group	RRID: AB_11183050; Cat#14927-1-AP
TIM23	Abcam	RRID: AB_10903878; Cat#ab116329
TIM44	Abcam	Cat#ab194829
TOM20	Cell Signaling	RRID: AB_2687663; Cat#D8T4N
TOM20	Santa Cruz Biotechnology	RRID: AB_2207533; Cat#sc-11415
TOM22	Abcam	RRID: AB_945897; ab57523
uL3m / MPRL3	Sigma-Aldrich	RRID: AB_2678606; Cat#HPA043665
uS15m / MRPS15	Proteintech Group	RRID: AB_2301068; Cat#17006-1-AP
uS17m / MRPS17	Proteintech Group	RRID: AB_10597844; Cat#18881-1-AP
VDAC	Abcam	RRID: AB_443084; Cat#ab14734
α-Tubulin	Sigma-Aldrich	RRID: AB_477583; Cat#T6199
Anti-rabbit IgG F(ab’)_2_-HRP	GE Healthcare	RRID: AB_772191; Cat#NA9340
Sheep Anti-Mouse IgG, Whole Ab ECL Antibody, HRP conjugated	GE Healthcare	RRID: AB_772209; Cat#NXA931
Donkey anti-Goat IgG (H+L) Cross-Adsorbed Secondary Antibody, HRP conjugated	Thermo Fisher	RRID: AB_2534679; Cat#A16005
Alexa Fluor 488 donkey anti-mouse antibody	Jackson Immuno	RRID: AB_2340850; Cat#715-546-151
Cy3-AffiniPure Donkey Anti-Rabbit IgG (H+L) antibody	Jackson Immuno	RRID: AB_2307443; Cat#711-165-152
F(ab)2-Goat anti-Mouse IgG (H+L) Cross-Adsorbed Secondary Antibody, Alexa Fluor 488	Thermo Fisher	RRID: AB_2534084; Cat#A-11017
Goat anti-Rabbit IgG (H+L) Highly Cross-Adsorbed Secondary Antibody, Alexa Fluor 568	Thermo Fisher	RRID: AB_10563566; Cat#A-11036

**Chemicals, Peptides, and Recombinant Proteins**		

2-chloroacetamid	Merck	Cat#8024120100
Anti-FLAG M2 beads	Sigma-Aldrich	Cat#A2220
Complete EDTA-free protease inhibitor cocktail	Roche	Cat#05056489001
Digitonin	Calbiochem	Cat#300410
Doxycycline	Sigma-Aldrich	Cat#D9891
Empore Octadecyl C18 Extraction Disks	3M	N/A
FLAG peptide	Sigma-Aldrich	Cat#F3290
FLAG® Immunoprecipitation Kit lysis buffer	Sigma-Aldrich	Cat#L3412
Guanidinium chloride	Sigma-Aldrich	Cat#G3272
Guanosine-5′-[β,γ-imido]triphosphate (GDPNP)	Jena Bioscience	Cat#NU-401-50
L-[^35^S]-methionine	Hartmann Analytic	Cat#SCM-01
Lipofectamine RNAiMAX transfection reagent	Invitrogen/Thermo Fisher	Cat#1377815
Lipofectamine2000 Transfection Reagent	Invitrogen/Thermo Fisher	Cat#11668027
Lipofectamine3000 Transfection Reagent	Invitrogen/Thermo Fisher	Cat#L3000008
Phenylmethylsulfonyl fluoride	Sigma-Aldrich	Cat#P7626
RNase inhibitor	New England Biolabs	Cat#M0307L
Tetracycline	Sigma-Aldrich	Cat#T7760
Tris(2-carboxyethyl)phosphine	Thermo Fisher	Cat#T2556
Triton X-100	Sigma-Aldrich	Cat#T8787
Trypsin Gold	Promega	Cat#V5280
α-[^32^P]-cytidine triphosphate	Hartmann Analytic	Cat#SRP-209
α-[^32^P]-deoxycytidine triphosphate	PerkinElmer	Cat#NEG513H
α-[^32^P]-uridine triphosphate	PerkinElmer	Cat#NEG007H
γ-[^32^P]-adenosine triphosphate	Hartmann Analytic	Cat#SRP-301

**Critical Commercial Assays**		

FLAG-immunoprecipitation kit	Sigma-Aldrich	Cat#FLAGIPT1
Prime-It II Random Primer Labeling Kit	Agilent	Cat#300385
Qubit Protein Assay Kit	Thermo Fisher	Cat#Q33211
Riboprobe® System - SP6/T7	Promega	Cat#P1460

**Experimental Models: Cell Lines**		

HeLa Cell Line *Homo sapiens*	ATCC Inc.	RRID: CVCL_0030
Flp-In T-REx HEK293 Cell Line *Homo sapiens*	Invitrogen/Thermo Fisher	RRID: CVCL_U427
Flp-In T-REx HEK293 Cell Line *GTPBP10* knock-out *Homo sapiens*	This study	N/A
Flp-In T-REx HEK293 Cell Line *PUSL1* knock-out *Homo sapiens*	This study	N/A
Flp-In T-REx HEK293 *GTPBP10*-Flag expression Cell Line *Homo sapiens*	This study	N/A
Flp-In T-REx HEK293 *PUSL1*-Flag expression Cell Line *Homo sapiens*	This study	N/A

**Experimental Models: Organisms/Strains**		

C57BL/6N control / wild-type mice	The Jackson Laboratory	RRID: MGI:5795896
*Mus musculus* C57BL/6N Genotype: *WT*^*+/+*^		
Heterozygous MitoRibo-Tag mice (*mL62-Flag* knock-in) *Mus musculus* C57BL/6N	This study	N/A
Genotype: *mL62-Flag*^*+/T*^		
MitoRibo-Tag mice (*mL62-Flag* knock-in)	This study	N/A
*Mus musculus* C57BL/6N		
Genotype: *mL62-Flag*^*T/T*^		
MitoRibo-Tag *Mterf4* control mice	This study	N/A
*Mus musculus* C57BL/6N		
Genotype: *mL62-Flag*^*T/T*^*Mterf4 loxP*^*P/P*^*Ckmm Cre*^*+/+*^		
MitoRibo-Tag *Mterf4* knock-out mice	This study	N/A
*Mus musculus* C57BL/6N		
Genotype:		
*mL62-Flag*^*T/T*^*Mterf4 loxP*^*P/P*^*Ckmm Cre*^*+/Cre*^		

**Oligonucleotides**		

See Table S3	See Table S3	See Table S3

**Recombinant DNA**		

pcDNA5/FRT/TO	Invitrogen/Thermo Fisher	Cat#V652020
pcDNA5/FRT/TO-GTPBP10-FLAG	This study	N/A
pcDNA5/FRT/TO-PUSL1-FLAG	This study	N/A
pcDNA5/FRT/TO-Mito-GFP	This study	N/A
pOG44 Flp-Recombinase Expression Vector	Invitrogen/Thermo Fisher	Cat#V600520
pSpCas9(BB)-2A-Puro (PX459) V2.0	Ran et al., 2013	RRID: Addgene_62988

**Software and Algorithms**		

cowplot version 0.9.2	R project	https://cran.r-project.org/web/packages/cowplot/index.html
CRISPOR	Haeussler et al., 2016	RRID: SCR_015935; http://www.crispor.tefor.net/
GraphPad Prism version 5	GraphPad Software	RRID: SCR_002798; https://www.graphpad.com/scientific-software/prism/
limma version 3.34.5	Ritchie et al., 2015	https://bioconductor.org/packages/3.3/bioc/html/limma.html
MaxQuant version 1.6.1.0	Cox and Mann, 2008	RRID: SCR_014485; http://www.coxdocs.org/doku.php?id=:maxquant:start
MitoProt	Claros and Vincens, 1996	https://ihg.gsf.de/ihg/mitoprot.html
R: A language and environment for statistical computing version 3.4.3	R Development Core Team, 2008	RRID: SCR_001905; http://www.R-project.org/
readxl version 1.1.0		https://cran.r-project.org/web/packages/readxl/index.html
Rstudio version 1.1.383	RStudio	RRID: SCR_000432; https://rstudio.com/
tidyverse version 1.2.1		https://cran.r-project.org/web/packages/tidyverse/index.html
TMHMM Server version 2.0	Sonnhammer et al., 1998	RRID: SCR_014935; http://www.cbs.dtu.dk/services/TMHMM/
Uniprot	Bateman et al., 2017	RRID:SCR_002380; http://www.uniprot.org
vsn version 3.46.0	Huber et al., 2002	RRID:SCR_001459; http://www.bioconductor.org/packages/release/bioc/html/vsn.html

**Other**		

Human reference proteome	Bateman et al., 2017	Uniprot: UP000005640
Mouse reference proteome	Bateman et al., 2017	Uniprot: UP000000589
Silencer select© negative control siRNA	Invitrogen/Thermo Fisher	Cat#4390844
Silencer select© siRNA *PUSL1*-siRNA1	Invitrogen/Thermo Fisher	Cat#s43084
Silencer select© siRNA *PUSL1-*siRNA2	Invitrogen/Thermo Fisher	Cat#s225579
ssniff M-Z Low-Phytoestrogen (mouse food newly weaned animals)	Ssniff Spezialdiaeten GmbH	N/A
ssniff RM-H Low-Phytoestrogen (mouse food)	Ssniff Spezialdiaeten GmbH	N/A

### Lead Contact and Materials Availability

Further information and requests for resources and reagents, which may require a completed Materials Transfer Agreement, should be directed to and will be fulfilled by the Lead Contact, Nils-Göran Larsson (nils-goran.larsson@ki.se).

### Experimental Model and Subject Details

#### Cell Lines

HeLa (RRID: CVCL_0030, female, ATCC), Flp-In T-REx HEK293 (HEK293T, RRID: CVCL_U427, female) and derivative cell lines (listed in the Key Resources Table) were grown in DMEM+GlutaMAX culture media (Thermo Fisher, cat. no. 31966-021) supplemented with 10% fetal bovine serum (Thermo Fisher, cat. no. 10270-106), 1x non-essential amino acids (Thermo Fisher, GIBCO, cat. no. 11140050), 100 U/ml Penicillin / 100 μg/ml Streptomycin (Thermo Fisher, GIBCO, cat. no. 15140122) and maintained at 37°C and 5% CO_2_. One day after thawing, culture media was refreshed to remove residual DMSO. Cells were split every second to fourth day and maintained at least one week before experiments were performed.

#### Generation of Knock-out Cell Lines

HEK293T *GTPBP10* and *PUSL1* knock-out cell lines were generated according to Ran et al. (2013) using the pSpCas9(BB)-2A-Puro (pX459) V2.0 vector (Ran et al., 2013), whereby gRNAs to edit PUSL1 were designed using the CRISPOR software (Haeussler et al., 2016). Essentially, the gRNAs were cloned into the pX459 vector which was transfected into cultured cells followed by puromycin selection (Ran et al., 2013). Two pairs of gRNAs targeting exon two were generated to induce an out-of-frame deletion within the *GTPBP10* coding sequence. To generate *PUSL1* knock-out HEK293T cells, gRNAs targeting exon 1 or exon 3 were cloned separately into the pX459 vector. Cells were transfected with pX459 using Lipofectamine 3000 according to the manufacturer's instructions. Transfected clones were selected with puromycin at 1.5 to 2 μg/ml (final concentration) for 48 h. Subsequently, cells were diluted to single cell suspension and plated on 96 well plates. Single colonies cells were sequenced with Sanger sequencing to confirm the predicted out-of-frame deletion or frameshift mutation. Knock-out of *GTPBP10* and *PUSL1* in selected cloned was confirmed by western blot and sequencing.

#### Generation of Stable Cell Lines

Flp-In T-REx HEK293 expression cell lines were generated according to manufacturer’s instructions with some modifications (Thermo Fisher, cat. no. K6500-01). Essentially, Flp-In T-REx HEK293 host cells were split and 300.000 cells were seeded into a 6-well dish with a final volume of 1 mL DMEM+GlutaMAX culture media, supplemented with 10% Tet-approved FBS (Clontech, cat. no. 631106) and non-essential amino acids but without antibiotics. On the next day, the pcDNA5/FRT/TO vector harboring, the gene of interest, and the pOG44 vector, harboring the *FLP* recombinase, were co-transfected with Lipofectamine2000 or Lipofectamine3000 (Thermo Fisher, cat. no. 11668027 and L3000008) Transfection Reagent to generate HEK293T expression cell lines. Transfected cells were selected adding 15 μg/ml Blasticidin S and 100 μg/ml Hygromycin B to culture media. Approximately two to three weeks post transfection positive colonies appeared and single colonies were picked and expanded. Protein expression was induced adding doxycycline (Sigma-Aldrich, cat. no. D9891) or tetracycline (Sigma-Aldrich, cat. no. T7760) to a final concentration of 50 ng/ml to culture media 24 h prior experimental analysis according to manufacturer’s instructions.

#### Animal Models

The mouse models used in this study belong to the C57BL/6N strain (RRID: MGI:5795896, The Jackson Laboratory). To generate mL62-Flag knock-in mice, the *mL62*-Flag knock-in targeting vector was constructed using BAC clones from the C57BL/6J RPCIB-731 BAC library by Taconic Biosciences (Cologne, Germany). The targeting vector harbored exons three to six of *mL62*, the FLAG-tag DNA sequence (introduced after the last amino acid of exon six: …MTMD-DYKDDDDK), a puromycin resistance selection marker (PuroR) flanked by F3 recombination sites (downstream of the *mL62* 3′UTR) and a thymidine kinase (Tk) cassette. After transfection of a C57BL/6N embryonic stem cell line, homologous recombinant clones were isolated using positive PuroR and negative Tk selections. The PuroR cassette was removed by crossing heterozygous-targeted knock-in mice with transgenic mice ubiquitously expressing the *Flp* recombinase. The remaining F3 recombination site remained in a non-conserved region of the genome. Heterozygous *mL62-Flag*^*+/T*^ knock-in mice were backcrossed with C57BL/6N wild-type mice for several generations. The backcrossed heterozygous *mL62-Flag*^*+/T*^ knock-in mice were intercrossed to generate homozygous *mL62-Flag*^*T/T*^ knock-in mice, denoted MitoRibo-Tag mice (*T/T*), constitutively expressing the mL62-FLAG fusion protein controlled by the endogenous promoter. Approved animal experiments (see ethics statement below) were performed with female and male homozygous MitoRibo-Tag^*T/T*^ mice at an age of 16 to 20 weeks and corresponding age and sex C57BL/6N wild-type mice as controls. Furthermore, MitoRibo-Tag mice were intercrossed with homozygous carriers of the *Mterf4* loxP allele to generate *Mterf4*^*L/L*^*, mL62*^*T/T*^ mice (Cámara et al., 2011). In subsequent crossings, *Mterf4*^*L/L*^*, mL62*^*T/T*^ mice were intercrossed with heterozygous C57BL/6N mice expressing the *Cre* recombinase under the *ckmm* promotor (genotype: *+/Cre*). Ultimately, intercrossed animals with the genotypes *Mterf4*^*L/L*^*, +/+, mL62*^*T/T*^ (control genotype) and *Mterf4*^*L/L*^*, +/Cre, mL62*^*T/T*^ (knock-out genotype), which lack the assembly factor MTERF4 in heart and skeletal muscle, were used for approved animal experiments at an age of 18 to 20 weeks, or for colony propagation. No female *Mterf4*^*L/L*^*, +/Cre, mL62*^*T/T*^ knock-out animals were used for matings.

#### Ethics Statement and Animal Housing

The study, including all animal experiments, was approved by the Landesamt für Natur, Umwelt und Verbraucherschutz Nordrhein–Westfalen (reference numbers 84-02.04.2015.A103 and 84-02.50.15.004) and performed in accordance with the recommendations and guidelines of the Federation of European Laboratory Animal Science Associations (FELASA). The health status of the animals is specific pathogen-free according to the FELASA recommendations. Wild-type C57BL/6N and transgenic mice were housed in individually ventilated cages (45 × 29 × 12 cm) with a 12 h light/dark cycle and controlled environmental conditions of 22 ± 2°C and 50 + 10% relative humidity. The animals were fed *ad libitum* on a standard mouse food (ssniff RM-H Low-Phytoestrogen, Ssniff Spezialdiaeten GmbH) or an enhanced diet during breeding and for newly weaned mice (ssniff M-Z Low-Phytoestrogen, Ssniff Spezialdiaeten GmbH).

### Method Details

#### Isolation of Mitochondria from Mouse Tissues

Heart, liver and kidney were dissected from mice and washed two times with ice cold phosphate-buffered saline (PBS, Thermo Fisher, cat. no. 14190-094). Hearts were additionally washed once with mitochondrial isolation buffer (MIB: 310 mM sucrose, 10 mM Tris-HCl (Merck, cat. no. 108382) and 0.05% fatty acid-free BSA (w/v; Sigma-Aldrich, cat. not. A8806)). Tissues were cut and gently homogenized within 5 to 15 mL of MIB with a Potter S (Sartorius). The volume of heart and kidney was brought to 15 mL and of liver samples to 50 mL total volume prior centrifugation for 10 min/1000 g/4°C to remove cell debris. The obtained mitochondria-containing supernatants from heart were centrifuged for 15 min/4500 g/4°C and from kidney and liver for 10 min/10000 g/4°C to isolate mitochondria. The crude mitochondrial pellets were resuspended in MIB supplemented with 1x complete EDTA-free protease inhibitor cocktail (PIC; Roche, cat. no. 05056489001), aliquoted and snap frozen. The protein concentration was determined by means of Bradford assay using BSA as standard.

#### Mitoribosome Co-immunoprecipitation

Crude mitochondrial protein of 1 mg was pelleted (5 min/9200 g/4°C) and resuspended with 200 μl purification-lysis buffer (10 mM Tris-HCl pH 7.5, 100 mM potassium chloride (KCl, Merck, cat. no. 1.04936.1000), 20 mM magnesium dichloride (MgCl_2_, Merck, cat. no. M2670), 1x PIC), 1% digitonin (Calbiochem, cat. no. 300410, 2 g detergent/g protein)). The lysates were incubated for 20 min on ice and clarified by centrifugation for 45 min/9200 g/4°C. The resulting supernatants were diluted 1:10 with dilution buffer (10 mM Tris-HCl pH 7.5, 100 mM KCl, 20 mM MgCl_2_) and incubated for 2 h at 4°C with 100 μl anti-FLAG M2 beads (Sigma-Aldrich, cat. no. A2220, equilibrated twice with 15 volumes TBS and wash buffer ( = dilution buffer supplemented with 0.1% digitonin)) while rotating at 10 rpm. Following binding, beads were washed three times with 15 volumes of wash buffer and two times with dilution buffer. Proteins were eluted with 300 μl elution buffer (50 mM Tris-HCl pH 7.5, 1 mM Tris(2-carboxyethyl)phosphine (TCEP; Thermo Fisher, cat. no. T2556), 5 mM 2-chloroacetamid (Merck, cat. no. 8024120100) and 1 ng/μl trypsin Gold (Promega, cat. no. V5280, resuspended in 50 mM acetic acid)) rotating for 30 min/RT for protein analysis or 60 min/4°C for RNA analysis rotating at 5 rpm. Eluates were transferred to ice and subsequently prepared for mass spectrometry or mixed 3:1 with TRIzol LS reagent (Thermo Fisher, cat. no. 10296028) for RNA analysis.

#### Mitoribosome Gradient Analysis

Sucrose density gradient fractionation of mitochondrial ribosomes was performed according to Matthews et al., (1982) and Rackham et al. (2016). Mitochondria (1 mg from mouse tissue or 750 μg from cultured cells) were re-isolated by centrifugation for 5 min/9200 g/4°C. Mitochondria were resuspended at protein concentrations of 7.5 mg/ml (from cultured cells) or 10 mg/ml (from mouse tissues) in lysis buffer (260 mM sucrose, 10 mM Tris-HCl pH 7.5, 100 mM KCl, 20 mM MgCl_2_, 2% digitonin for mouse mitochondria or 6% digitonin for human mitochondria, 40 U/ml RNase inhibitor (New England Biolabs, cat. no. M0307L) and 1x PIC) and incubated for 20 min on ice. Samples were clarified by centrifugation for 45 min/9200 g/4°C. Sucrose density gradients of 10% to 30% were cast as described by Metodiev et al. (2009) using a Gradient Master (BioComp Instruments, Inc.). The sucrose gradient solutions were prepared with 10 mM Tris-HCl pH 7.5, 100 mM KCl and 20 mM MgCl_2_. Ten percent of the cleared mitochondrial lysates were saved as input control. The remaining lysate was loaded on top of the 10% - 30% sucrose density gradient and centrifuged for 15 h/71092 g/4°C. Fractions were collected from the top (first fraction: 750 μl and loaded lysate volume, fraction two to 15: 750 μl and fraction 16: residual volume). The input samples and one third of each fraction were precipitated with 0.02% sodium deoxycholate and 12% trichloroacetic acid (final concentrations) for SDS-PAGE and western blot analysis. The loaded input fractions of mitochondrial lysate from mouse correspond to ∼30% of total and the input fraction of mitochondrial lysates from human cultured cells to ∼16% of total. Mitoribosomal proteins were detected using antibodies listed in the STAR methods table. Chemiluminescent detection was carried out with Amersham ECL Western Blotting Detection reagent (GE Healthcare, cat. no. RPN2106).

#### Preparation of Mitochondria for Proteomics

Mitochondria were resuspended with an appropriate volume of guanidinium hydrochloride lysis buffer (6 M guanidinium chloride (Sigma-Aldrich, cat. no. G3272), 10 mM TCEP, 40 mM chloroacetamide and 100 mM Tris-HCl) as previously described by Kulak et al., 2014 with some modifications. Lysates were diluted 1:10 with 20 mM Tris pH 8.3 and digested overnight at 37°C with at least 300 ng or 1:200 (protein:trypsin) of trypsin Gold (Promega). Peptides were desalted using home-made StageTips (Empore Octadecyl C18, 3M; Rappsilber et al., 2003) and eluted with 80 to 100 μl of 60% acetonitrile / 0.1% formic acid buffer. The peptides were dried with a vacuum concentrator plus (Eppendorf) and resuspended with 0.1% formic acid for mass spectrometry.

#### Liquid-Chromatography Mass Spectrometry

Peptides obtained from co-immunoprecipitations were separated on a 25 cm, 75 μm internal diameter PicoFrit analytical column (New Objective) packed with 1.9 μm ReproSil-Pur 120 C18-AQ media (Dr. Maisch) using an EASY-nLC 1000 or EASY-nLC 1200 (Thermo Fisher Scientific). The column was maintained at 50°C. Buffer A and B were 0.1% formic acid in water and 0.1% formic acid in acetonitrile or 0.1% formic acid in 80% acetonitrile. Peptides were separated on a segmented gradient from 6% to 25% or 31% buffer B. Eluting peptides were analyzed on an Orbitrap Fusion or QExactive HF mass spectrometer (Thermo Fisher Scientific). Peptide precursor m/z measurements were carried out at 60000 resolution in the 300 to 1500 or 1800 m/z range. The ten most intense precursors with charge state from two to seven only were selected for HCD fragmentation using 27% or 25% normalized collision energy. The m/z values of the peptide fragments were measured in the orbitrap at a resolution of 30000 using an AGC target of 2e5 and 55, 80 or 100 ms maximum injection time. Upon fragmentation, precursors were put on a dynamic exclusion list for 45 s.

#### Mass Spectrometry Data Analysis

The raw data were analyzed with MaxQuant version 1.6.1.0 using the integrated Andromeda search engine (Cox and Mann, 2008, Cox et al., 2011). Peptide fragmentation spectra were searched against the canonical and isoform sequences of the mouse (Uniprot proteome ID: UP000000589, downloaded September 2018 from UniProt) or human (Uniprot proteome: ID UP000005640, downloaded September 2018 from UniProt) reference proteome (Bateman et al., 2017). Methionine oxidation and protein N-terminal acetylation were set as variable modifications; cysteine carbamidomethylation was set as fixed modification. The digestion parameters were set to “specific” and “Trypsin/P.” The minimum number of peptides and razor peptides for protein identification was one and the minimum number of unique peptides was zero. Protein identification was performed at a peptide spectrum matches and protein false discovery rate of 0.01. The “second peptide” option was on. Successful identifications were transferred between the different raw files using the “Match between runs” option. Label-free quantification (LFQ) was performed using an LFQ minimum ratio count of two. LFQ intensities were filtered for a number of valid values that was equal to the minimum number of replicates in an experimental group minus one. Missing values were imputed from a normal distribution with a width of 0.3 and down shift of 1.8. For the analysis of the label-free data from the MitoRibo-Tag CoIPs in heart, liver, and kidney mitochondria, the median enrichment value was added to all label-free intensities in order to remove the global shift resulting from the label-free normalization. MitoCarta annotations were added using the primary gene name and the first of the gene name synonyms of the oldest Uniprot ID with the highest number of peptides (Calvo et al., 2016, Pagliarini et al., 2008). For mitochondrial proteome comparisons from mouse heart, liver, and kidney only mitochondrial proteins, according to MitoCarta, were considered and the data were normalized using the R package variance stabilization (vsn; Huber et al., 2002, R Development Core Team, 2008). Differential abundance analysis was performed using limma (Ritchie et al., 2015).

#### SDS-Polyacrylamide Gel Electrophoresis

Protein samples were resuspended with 2x NuPAGE® LDS sample buffer (Thermo Fisher, cat. no. NP0007) supplemented with 100 mM dithiothreitol and separated on NuPAGE 4 – 12% or 10% Bis-Tris midi gels (Thermo Fisher, cat. no. WG1202BOX and WG1402BOX) using NuPAGE 1x MOPS (cat. no. NP0001) or 1x MES (cat. no. NP0002) running buffer for appropriate separation.

#### Western Blot

Proteins were blotted onto PVDF membrane using 1x wet western blot buffer (38.63 mM glycine, 47.87 mM Tris and 10% methanol) at 4°C for 2 h at 400 mA or overnight at 80 mA. Membranes were blocked for 1 h at RT with blocking buffer (Rockland, cat. no. MB-070) or 5% milk-Tris buffer saline (TBS; 50 mM Tris-HCl pH 7.4 and 150 mM NaCl). The blocked membranes were incubated overnight with primary antibodies at indicated dilutions. Following overnight incubation, membranes were washed three times with 1x TBS-Tween20 (TBST; TBS with 0.1% Tween20) and incubated with HRP-conjugated secondary antibodies for 1.5 to 2 h at RT. Membranes were washed two times with 1x TBST and 1x TBS. Detection was performed using Amersham ECL western blotting detection reagent (GE Healthcare, Amersham, cat. no. RPN2106).

#### Isolation of Genomic DNA from Murine Tissue

400 μl tissue lysis buffer (0.5% SDS (Merck, cat. no. 112533), 100 mM NaCl, 20 mM Tris pH 8, 2.5 mM EDTA pH 8 (Merck, cat. no. 108418)) was supplemented with 80 μg proteinase K and added to each ear clip or tail cut of a mouse. The samples were lyzed for 5 to 24 h at 56°C. Afterwards, 40 μl 8 M potassium acetate and subsequently 500 μl chloroform were added to each sample and DNA was pelleted by centrifugation for 10 min/13000 rpm/RT. The resulting upper aqueous phase was carefully transferred to a new tube and mixed with 1 mL of 95% ethanol. DNA was precipitated by incubation at −80°C for 30 min and pelleted by centrifugation for 15 min/13000 rpm/4°C. The resulting supernatant was discarded, and the DNA pellets were washed once with 500 μl of 70% ethanol. The DNA was subsequently dried for 15 min at 60°C using a vacuum concentrator plus (Eppendorf). Finally, the DNA was dissolved with 125 μl water and stored at 4°C for further use.

#### RNA Isolation

RNA was isolated using the Trizol (for RNA of cultured cells) or Trizol LS (for RNA obtained from co-immunoprecipitations of mouse mitochondria) reagents (Thermo Fisher, cat. no. (15596026 and 10296028) according to manufacturer’s instructions. RNA was precipitated overnight in the presence of glycogen.

#### Northern Blot

Isolated RNA of 1 to 4 μg were solubilized in NorthernMax*-*Gly Sample loading dye (Thermo Fisher, cat. no. AM8551) and loaded onto a 1.2% LE agarose gels. Following separation, gels were blotted overnight onto Hybond-N+ membrane (GE Healthcare, cat. no. 45-000-927) according to standard procedures. The probes for labeling of mouse and human mitochondrial 12S and 16S rRNA were prepared using the Prime-It II Random Primer labeling kit (Agilent) and 50 μCi α-[^32^P]-CTP (Hartmann Analytic, cat. no. SRP-209) for mouse rRNAs or α-[^32^P]-dCTP (PerkinElmer, cat. no. NEG513H). tRNA probes against mouse mitochondrial tRNA were prepared by T4 polynucleotide kinase end labeling using with 50 μCi γ-[^32^P]-ATP (Hartmann Analytic, cat. no. SRP-301). For labeling of human tRNAs 4 μg total RNA were loaded onto a 15% TBE-urea gel in 1x TBE buffer and wet transferred at 30 V for 1 hour in 0.5x TBE buffer. The tRNA probes against human mitochondrial tRNAs were prepared using the Riboprobe *in vitro* Transcription System (Promega) with 50 μCi α-[^32^P]-UTP (PerkinElmer, cat. no. NEG007H).

#### Cell Growth Assay

For growth measurements, 20.000 cells were seeded into 6-well plates and grown in the presence or absence of 50 ng/ml of doxycycline in glucose-free DMEM containing 0.9 g/l galactose, 10% (v/v) FBS, 2 mM Glutamax and 1 x Penicillin/Streptomycin. Cell confluence was determined every second day with EVE™ Automated Cell Counter (NanoEnTek, cat. no. E1000).

#### RNA Interference using siRNAs

HEK293T were cultured for several days and seeded 24 h before first transfection in DMEM+GlutaMAX culture media supplemented with 50 μg/ml uridine (Sigma-Aldrich, cat. no. U3003). For *in cellulo* translation experiments 300.000 control and 400.000 knock-down cells were seeded, whereas 1.5 million control and 3 million cells were seeded onto 500 cm^2^ square dishes for isolation of mitochondria (Thermo Fisher, cat. no. 10489282). On the next day, 2.875 μg (for 10 cm-dishes) or 18 μg siRNA (for square dishes) of silencer select© siRNA (Thermofisher, negative control: cat. no. 4390844, *PUSL1*-siRNA1: cat. no. s43084, *PUSL1-*siRNA2 cat. no. s225579) were transfected using 15 μl or 90 μl Lipofectamine RNAiMAX transfection reagent (Thermo Fisher, cat. no. 13778150) according to manufacturer’s instructions. On the third day, the siRNA transfection was repeated and cells were incubated for two to three additional days.

#### *In Cellulo* Translation Assay

HEK293T knock-out or knock-down and control cells were carefully washed with 3 mL PBS and incubated for 30 min in labeling media (DMEM, high glucose, no glutamine, no methionine, no cysteine, supplemented with 10% dialyzed fetal bovine serum (Thermo Fisher, cat. no. A3382001), 1x GlutaMax (Thermo Fisher, cat. no. 3505003), sodium pyruvate (Thermo Fisher, cat. no. 11360039) and 50 μg/ml uridine). Meanwhile, emetine (Sigma-Aldrich, cat. no. E2375) was solubilized in PBS, sterile filtered and added to a final concentration of 100 μg/ml, followed by 5 min incubation, to inhibit cytosolic translation. Subsequently, 1000 μCi L-[^35^S]-methionine (Hartmann Analytic, cat. no. SCM-01) were added to each dish and incubated for 30 min or 1 h at 37°C / 5% CO_2_. Following labeling, cells were washed three times with 5 mL PBS, transferred into 1.5 mL Eppendorf tubes, resuspended with 2x RIPA buffer (150 mM sodium chloride, 1% triton X-100, 0.5% sodium deoxycholate, 0.1% SDS (sodium dodecyl sulfate) and 50 mM Tris, pH 8 supplemented with 1x PIC)) and snap frozen. Cell lysates were thawed and sonicated three times for 20 s at 90% amplitude with a sonoplus mini20 (Bandelin, cat. no. 3665) equipped with a MS 1.5 sonotrode (Bandelin, cat. no. 3639). Insoluble material was removed by centrifugation for 5 min/2000 rpm/4°C. The resulting supernatants were saved and quantified using Qubit Protein Assay Kit (Thermo Fisher, cat. no. Q33211). An appropriate amount of protein lysate was mixed with 2x SDS sample buffer and 20 μg of lysate from each approach were loaded onto NuPAGE 12% Bis-Tris protein gels (ran with 1x MES buffer). Gels were subsequently incubated for 30 min in protein fixation buffer (20% ethanol and 10% acetic acid) supplemented with PhastGel Blue R (Sigma-Aldrich, cat. no. B4921) and scanned. Afterwards, gels were destained with fixation buffer and incubated for 30 min in Amersham Amplify (GE Healthcare, cat. no. NAMP100) to increase sensitivity of the radioactive signal. Detection was performed using a Typhoon FLA7000 (GE Healthcare).

#### Isolation of Mitochondria from Cultured Cells

Dishes with ∼80 to 90% confluent cells were harvested (from two to three 500 cm^2^ dishes). The cells were subsequently pelleted by centrifugation for 5 min/800 g/4°C and washed two times with cold PBS. The cell pellet was initially resuspended with 2 mL isolation buffer (20 mM HEPES pH 7.6, 220 mM mannitol, 20 mM sucrose, 1 mM EDTA, 2 mg/ml fatty acid-free BSA and 1x PIC). Thereafter, 6 mL isolation buffer was added to the samples followed by 15 min incubation on ice to facilitate cell swelling. Swollen cells were gently homogenized with 20x strokes at 500 rpm using a Schuett homgenplus (Schuett Biotec, cat. no. 3.201-011). The cell homogenates were centrifuged for 5 min/800 g/4°C to remove cell debris. The resulting supernatant was centrifuged for 10 min/10000 g/4°C to obtain a crude mitochondrial pellet, which was washed two times with isolation buffer without BSA prior protein quantification. For some experiments mitochondria from cultured cells were isolated according Lee and Bogenhagen (2016) with some modifications as described below. All buffers were prepared with DEPC-treated water. The mitochondrial pellet obtained after the nuclease treatment (step 12 of the medium/large-scale preparation from Lee and Bogenhagen [2016]) was washed twice with 1 mL of cell mitochondrial isolation buffer, aliquoted, snap frozen and stored at −80°C.

#### Protein Co-immunoprecipitation

Co-immunoprecipitations of putative MIPs were performed with mitochondria isolated from HEK293T cells. Essentially, 750 μg of mitochondria were lyzed with 150 μl purification-lysis buffer supplemented with 3% digitonin (for purification of PUSL1-FLAG-associated complexes) or FLAG® immunoprecipitation kit lysis buffer (Sigma-Aldrich, cat. no. L3412) supplemented with 1% triton X-100 (for purification of GTPBP10-FLAG-associated complexes) and 5 mM MgCl_2_. All lysis buffers were additionally supplemented with 1x PIC. At indicated experiments guanosine-5′-[β,γ-imido]triphosphate (Jena Bioscience, cat. no. NU-401-50) was added to a final concentration of 20 mM to the lysis buffer. Protein complexes were subsequently purified as described in “Mitoribosome co-immunoprecipitation from mouse mitochondria” or using the FLAG-immunoprecipitation kit (Sigma-Aldrich, cat. no. FLAGIPT1) with buffers freshly supplemented with 5 mM MgCl_2_. Elution of GTPBP10-FLAG co-immunoprecipitations carried out using the FLAG peptide at 300 ng/μl final concentration (Sigma-Aldrich, cat. no. F3290) according to manufacturer’s instructions.

#### Immunocytochemistry and Microscopy

30.000 HeLa cells were seeded onto coverslips in 12 well-dish plates. One day after seeding, cells were transiently transfected with 1 μg of vector DNA, harboring the gene-of-interest, and 0.5 μl Lipofectamine2000 per well according to manufacturer’s instructions. Following 24 h incubation, cells were fixed with 4% PFA (EM sciences, cat. no. 15710) in PBS for 10 min at RT. After several washes with PBS, cells were permeabilized with 0.1% triton X-100 in PBS for 5 min at RT. Unspecific sites were blocked with 3% BSA (Sigma-Aldrich, cat. no. A3858) in PBS. Primary and secondary antibodies were also prepared with 3% BSA. The following primary antibodies were used and incubated at 4°C overnight, mouse anti-Flag (1:500, Cell Signaling, cat. no. 8146) and rabbit anti-TOM20 (1:1000, Santa Cruz Biotechnology, cat. no. 11415). After several washes with 3% BSA, the following secondary antibodies were applied for two hours at RT: donkey anti-mouse conjugated to Alexa Fluor 488 nm (Jackson Immuno, cat. no. 715-546-151) and donkey anti-rabbit conjugated to Cy3 (Jackson Immuno, cat. no. 711-165-152). Coverslips were washed with PBS and nuclei counterstained with DAPI before mounting with Aqua-Poly/Mount (Polysciences, cat. no. 18606). A laser-scanning confocal microscope (Leica, TCS SP8-X) equipped with a white light laser, a 405-diode UV laser, a 100x objective lens (HCX Plan-Apochromat CS 100x oil, 1.46 NA), and hybrid detectors was used to acquire fluorescence images. The following parameters were used for acquisition: image size of 1,024 × 1,024 pixels, bi-directional X, scan speed 200 Hz, and z-step size 0.2 μm.

#### Sodium Carbonate Extraction of Proteins

For each approach, 200 μg of crude mitochondria were initially resuspended in 100 μl mitochondrial isolation buffer and sonicated on ice, whereas one aliquot of mitochondria was kept on ice as total protein control. The sonicated mitochondria were pelleted and subsequently resuspended in 200 μl of 20 mM HEPES-KOH pH 7.4 supplemented with 1x PIC. After the initial resuspension, additional 200 μl of HEPES buffer or 200 μl of 200 mM sodium carbonate buffer (Na_2_CO_3_; Sigma-Aldrich, cat. no. S7795) at pH 10.5, 11. 5 or 12.5, were added to the samples, followed by gently mixing and 30 min incubation on ice. The samples were then centrifuged for 30 min/45000 rpm/4°C to separate soluble and membrane (-associated) proteins. The supernatants were precipitated in the presence of 0.02% sodium deoxycholate with 12% trichloroacetic acid and further analyzed by SDS-PAGE and western blot.

#### Protease Protection Assay

Freshly prepared mitochondria (200 μg) for each approach were pelleted and resuspended in 200 μl isolation buffer, hypotonic swelling buffer (10 mM HEPES-KOH, pH 7.4) or isolation buffer supplemented with 1% triton X-100. The aliquots were split into two, whereby to one aliquot proteinase K (Roche, cat. no. 03450384103) was added to a final concentration of 25 μg/ml. Samples were incubated on ice for 10 min and subsequently inhibited with 2 μl of 0.1 M phenylmethylsulfonyl fluoride (PMSF, Sigma-Aldrich, cat. no. P7626) added to each sample. Proteins were precipitated with 0.02% sodium deoxycholate and 12% trichloroacetic acid and further analyzed by SDS-PAGE and western blot.

### Quantification and Statistical Analysis

Quantitative proteomics data (Related to Figures 3, 4, 5, S1, S2, S3, S4, and S6) were analyzed using limma (Ritchie et al., 2015). The statistical significance of differential expression or protein enrichment analysis was calculated using limma’s moderated t test. The number of experiments or (biological) replicates (n) used for the statistical evaluation of each experiment are indicated in corresponding figure legends, whereby the significance is represented by the adjusted p value using the Benjamini and Hochberg method (Benjamini and Hochberg, 1995). The adjusted p values, in addition to the log fold change values and the 95% confidence interval values for the log fold change are reported in the supplemental data Tables S1 and S2. In Figure S4D, the mean growth of each cell line was determined based on three replicates and analyzed using GraphPad Prism version 5 (GraphPad Software) applying a two-way analysis of variance test (ANOVA) and the Bonferroni correction (p > 0.05 not significant; p < 0.0001 significant). The error bars represent the standard error of the mean (from three replicate experiments).

### Data and Code Availability

The published article includes the mass spectrometry datasets generated and analyzed during this study, which can be found in the supplemental data tables Table S1 – Label-free quantitative mass spectrometry data and Table S2 – Quantification of label-free quantitative mass spectrometry data.

## Supplemental Information

Document S1. Figures S1–S6 and Table S3

Table S1. Label-free Quantitative Mass Spectrometry Data, Related to Figures 3, 4, 5, S1, S2, S3, S4, and S6

Table S2. Quantification of Label-free Quantitative Mass Spectrometry Data, Related to Figures 3, 4, 5, S1, S2, S3, S4, and S6

Document S2. Article plus Supplemental Information

## References

[bib1] Akabane S., Ueda T., Nierhaus K.H., Takeuchi N. (2014). Ribosome rescue and translation termination at non-standard stop codons by ICT1 in mammalian mitochondria. PLoS Genet..

[bib2] Amunts A., Brown A., Toots J., Scheres S.H.W., Ramakrishnan V. (2015). Ribosome. The structure of the human mitochondrial ribosome. Science.

[bib3] Antonicka H., Sasarman F., Nishimura T., Paupe V., Shoubridge E.A. (2013). The mitochondrial RNA-binding protein GRSF1 localizes to RNA granules and is required for posttranscriptional mitochondrial gene expression. Cell Metab..

[bib4] Antonicka H., Choquet K., Lin Z.Y., Gingras A.C., Kleinman C.L., Shoubridge E.A. (2017). A pseudouridine synthase module is essential for mitochondrial protein synthesis and cell viability. EMBO Rep..

[bib5] Arroyo J.D., Jourdain A.A., Calvo S.E., Ballarano C.A., Doench J.G., Root D.E., Mootha V.K. (2016). A Genome-wide CRISPR Death Screen Identifies Genes Essential for Oxidative Phosphorylation. Cell Metab..

[bib6] Bateman A., Martin M.J., O’Donovan C., Magrane M., Alpi E., Antunes R., Bely B., Bingley M., Bonilla C., Britto R., The UniProt Consortium (2017). UniProt: the universal protein knowledgebase. Nucleic Acids Res..

[bib7] Benjamini Y., Hochberg Y. (1995). Controlling the False Discovery Rate: A Practical and Powerful Approach to Multiple Testing. J. R. Stat. Soc. B.

[bib8] Bogenhagen D.F., Martin D.W., Koller A. (2014). Initial steps in RNA processing and ribosome assembly occur at mitochondrial DNA nucleoids. Cell Metab..

[bib9] Bogenhagen D.F., Ostermeyer-Fay A.G., Haley J.D., Garcia-Diaz M. (2018). Kinetics and Mechanism of Mammalian Mitochondrial Ribosome Assembly. Cell Rep..

[bib10] Brown A., Rathore S., Kimanius D., Aibara S., Bai X.C., Rorbach J., Amunts A., Ramakrishnan V. (2017). Structures of the human mitochondrial ribosome in native states of assembly. Nat. Struct. Mol. Biol..

[bib11] Calvo S.E., Clauser K.R., Mootha V.K. (2016). MitoCarta2.0: an updated inventory of mammalian mitochondrial proteins. Nucleic Acids Res..

[bib12] Cámara Y., Asin-Cayuela J., Park C.B., Metodiev M.D., Shi Y., Ruzzenente B., Kukat C., Habermann B., Wibom R., Hultenby K. (2011). MTERF4 regulates translation by targeting the methyltransferase NSUN4 to the mammalian mitochondrial ribosome. Cell Metab..

[bib13] Claros M.G., Vincens P. (1996). Computational method to predict mitochondrially imported proteins and their targeting sequences. Eur. J. Biochem..

[bib14] Cox J., Mann M. (2008). MaxQuant enables high peptide identification rates, individualized p.p.b.-range mass accuracies and proteome-wide protein quantification. Nat. Biotechnol..

[bib15] Cox J., Neuhauser N., Michalski A., Scheltema R.A., Olsen J.V., Mann M. (2011). Andromeda: a peptide search engine integrated into the MaxQuant environment. J. Proteome Res..

[bib16] Davis J.H., Williamson J.R. (2017). Structure and dynamics of bacterial ribosome biogenesis. Philos. Trans. R. Soc. Ser. B Biol. Sci..

[bib17] Del Campo M., Ofengand J., Malhotra A. (2004). Crystal structure of the catalytic domain of RluD, the only rRNA pseudouridine synthase required for normal growth of Escherichia coli. RNA.

[bib18] Dennerlein S., Rozanska A., Wydro M., Chrzanowska-Lightowlers Z.M.A., Lightowlers R.N. (2010). Human ERAL1 is a mitochondrial RNA chaperone involved in the assembly of the 28S small mitochondrial ribosomal subunit. Biochem. J..

[bib19] Greber B.J., Ban N. (2016). Structure and Function of the Mitochondrial Ribosome. Annu. Rev. Biochem..

[bib20] Greber B.J., Bieri P., Leibundgut M., Leitner A., Aebersold R., Boehringer D., Ban N. (2015). The complete structure of the 55S mammalian mitochondrial ribosome. Science.

[bib21] Guarani V., Paulo J., Zhai B., Huttlin E.L., Gygi S.P., Harper J.W. (2014). TIMMDC1/C3orf1 functions as a membrane-embedded mitochondrial complex I assembly factor through association with the MCIA complex. Mol. Cell. Biol..

[bib22] Haeussler M., Schönig K., Eckert H., Eschstruth A., Mianné J., Renaud J.-B., Schneider-Maunoury S., Shkumatava A., Teboul L., Kent J. (2016). Evaluation of off-target and on-target scoring algorithms and integration into the guide RNA selection tool CRISPOR. Genome Biol..

[bib23] Hällberg B.M., Larsson N.G. (2014). Making proteins in the powerhouse. Cell Metab..

[bib24] Haque M.E., Elmore K.B., Tripathy A., Koc H., Koc E.C., Spremulli L.L. (2010). Properties of the C-terminal tail of human mitochondrial inner membrane protein Oxa1L and its interactions with mammalian mitochondrial ribosomes. J. Biol. Chem..

[bib25] Heublein M., Burguillos M.A., Vögtle F.N., Teixeira P.F., Imhof A., Meisinger C., Ott M. (2014). The novel component Kgd4 recruits the E3 subunit to the mitochondrial α-ketoglutarate dehydrogenase. Mol. Biol. Cell.

[bib26] Huber W., von Heydebreck A., Sültmann H., Poustka A., Vingron M. (2002). Variance stabilization applied to microarray data calibration and to the quantification of differential expression. Bioinformatics.

[bib27] Hur S., Stroud R.M. (2007). How U38, 39, and 40 of many tRNAs become the targets for pseudouridylation by TruA. Mol. Cell.

[bib28] Jackson C.B., Huemer M., Bolognini R., Martin F., Szinnai G., Donner B.C., Richter U., Battersby B.J., Nuoffer J.-M., Suomalainen A., Schaller A. (2019). A variant in MRPS14 (uS14m) causes perinatal hypertrophic cardiomyopathy with neonatal lactic acidosis, growth retardation, dysmorphic features and neurological involvement. Hum. Mol. Genet..

[bib29] Jourdain A.A., Koppen M., Wydro M., Rodley C.D., Lightowlers R.N., Chrzanowska-Lightowlers Z.M., Martinou J.C. (2013). GRSF1 regulates RNA processing in mitochondrial RNA granules. Cell Metab..

[bib30] Kehrein K., Schilling R., Möller-Hergt B.V., Wurm C.A., Jakobs S., Lamkemeyer T., Langer T., Ott M. (2015). Organization of Mitochondrial Gene Expression in Two Distinct Ribosome-Containing Assemblies. Cell Rep..

[bib31] Keilhauer E.C., Hein M.Y., Mann M. (2015). Accurate protein complex retrieval by affinity enrichment mass spectrometry (AE-MS) rather than affinity purification mass spectrometry (AP-MS). Mol. Cell. Proteomics.

[bib32] Kim H.-J., Barrientos A. (2018). MTG1 couples mitoribosome large subunit assembly with intersubunit bridge formation. Nucleic Acids Res..

[bib33] Kolanczyk M., Pech M., Zemojtel T., Yamamoto H., Mikula I., Calvaruso M.-A., van den Brand M., Richter R., Fischer B., Ritz A. (2011). NOA1 is an essential GTPase required for mitochondrial protein synthesis. Mol. Biol. Cell.

[bib34] Kühl I., Miranda M., Atanassov I., Kuznetsova I., Hinze Y., Mourier A., Filipovska A., Larsson N.G. (2017). Transcriptomic and proteomic landscape of mitochondrial dysfunction reveals secondary coenzyme Q deficiency in mammals. eLife.

[bib35] Kulak N.A., Pichler G., Paron I., Nagaraj N., Mann M. (2014). Minimal, encapsulated proteomic-sample processing applied to copy-number estimation in eukaryotic cells. Nat. Methods.

[bib36] Lavdovskaia E., Kolander E., Steube E., Mai M.M.-Q., Urlaub H., Richter-Dennerlein R. (2018). The human Obg protein GTPBP10 is involved in mitoribosomal biogenesis. Nucleic Acids Res..

[bib37] Lee K.W., Bogenhagen D.F. (2016). Scalable Isolation of Mammalian Mitochondria for Nucleic Acid and Nucleoid Analysis. Methods Mol. Biol..

[bib38] Lee R.G., Rudler D.L., Rackham O., Filipovska A. (2018). Is mitochondrial gene expression coordinated or stochastic?. Biochem. Soc. Trans..

[bib39] Liu M., Spremulli L. (2000). Interaction of mammalian mitochondrial ribosomes with the inner membrane. J. Biol. Chem..

[bib40] Lorenzi I., Oeljeklaus S., Aich A., Ronsör C., Callegari S., Dudek J., Warscheid B., Dennerlein S., Rehling P. (2018). The mitochondrial TMEM177 associates with COX20 during COX2 biogenesis. Biochim. Biophys. Acta Mol. Cell. Res..

[bib41] Maiti P., Kim H.-J., Tu Y.-T., Barrientos A. (2018). Human GTPBP10 is required for mitoribosome maturation. Nucleic Acids Res..

[bib42] Matthews D.E., Hessler R.A., Denslow N.D., Edwards J.S., O’Brien T.W. (1982). Protein composition of the bovine mitochondrial ribosome. J. Biol. Chem..

[bib43] Metodiev M.D., Lesko N., Park C.B., Cámara Y., Shi Y., Wibom R., Hultenby K., Gustafsson C.M., Larsson N.G. (2009). Methylation of 12S rRNA is necessary for in vivo stability of the small subunit of the mammalian mitochondrial ribosome. Cell Metab..

[bib44] Metodiev M.D., Spåhr H., Loguercio Polosa P., Meharg C., Becker C., Altmueller J., Habermann B., Larsson N.G., Ruzzenente B. (2014). NSUN4 is a dual function mitochondrial protein required for both methylation of 12S rRNA and coordination of mitoribosomal assembly. PLoS Genet..

[bib45] Mick D.U., Vukotic M., Piechura H., Meyer H.E., Warscheid B., Deckers M., Rehling P. (2010). Coa3 and Cox14 are essential for negative feedback regulation of COX1 translation in mitochondria. J. Cell Biol..

[bib46] Mick D.U., Dennerlein S., Wiese H., Reinhold R., Pacheu-Grau D., Lorenzi I., Sasarman F., Weraarpachai W., Shoubridge E.A., Warscheid B., Rehling P. (2012). MITRAC links mitochondrial protein translocation to respiratory-chain assembly and translational regulation. Cell.

[bib47] Mitchell A.L., Attwood T.K., Babbitt P.C., Blum M., Bork P., Bridge A., Brown S.D., Chang H.-Y., El-Gebali S., Fraser M.I. (2019). InterPro in 2019: improving coverage, classification and access to protein sequence annotations. Nucleic Acids Res..

[bib48] Möller-Hergt B.V., Carlström A., Stephan K., Imhof A., Ott M. (2018). The ribosome receptors Mrx15 and Mba1 jointly organize cotranslational insertion and protein biogenesis in mitochondria. Mol. Biol. Cell.

[bib49] Mootha V.K., Bunkenborg J., Olsen J.V., Hjerrild M., Wisniewski J.R., Stahl E., Bolouri M.S., Ray H.N., Sihag S., Kamal M. (2003). Integrated analysis of protein composition, tissue diversity, and gene regulation in mouse mitochondria. Cell.

[bib50] O’Brien T.W., Kalf G.F. (1967). Ribosomes from rat liver mitochondria. I. Isolation procedure and contamination studies. J. Biol. Chem..

[bib51] O’Brien T.W., Kalf G.F. (1967). Ribosomes from rat liver mitochondira. II. Partial characterization. J. Biol. Chem..

[bib52] Ofengand J. (2002). Ribosomal RNA pseudouridines and pseudouridine synthases. FEBS Lett..

[bib53] Ott M., Prestele M., Bauerschmitt H., Funes S., Bonnefoy N., Herrmann J.M. (2006). Mba1, a membrane-associated ribosome receptor in mitochondria. EMBO J..

[bib54] Ott M., Amunts A., Brown A. (2016). Organization and Regulation of Mitochondrial Protein Synthesis. Annu. Rev. Biochem..

[bib55] Pagliarini D.J., Calvo S.E., Chang B., Sheth S.A., Vafai S.B., Ong S.E., Walford G.A., Sugiana C., Boneh A., Chen W.K. (2008). A mitochondrial protein compendium elucidates complex I disease biology. Cell.

[bib56] Perks K.L., Rossetti G., Kuznetsova I., Hughes L.A., Ermer J.A., Ferreira N., Busch J.D., Rudler D.L., Spahr H., Schöndorf T. (2018). PTCD1 Is Required for 16S rRNA Maturation Complex Stability and Mitochondrial Ribosome Assembly. Cell Rep..

[bib57] R Development Core Team (2008). R: A language and environment for statistical computing.

[bib58] Rackham O., Busch J.D., Matic S., Siira S.J., Kuznetsova I., Atanassov I., Ermer J.A., Shearwood A.M.J., Richman T.R., Stewart J.B. (2016). Hierarchical RNA Processing Is Required for Mitochondrial Ribosome Assembly. Cell Rep..

[bib59] Ran F.A., Hsu P.D., Wright J., Agarwala V., Scott D.A., Zhang F. (2013). Genome engineering using the CRISPR-Cas9 system. Nat. Protoc..

[bib60] Rappsilber J., Ishihama Y., Mann M. (2003). Stop and go extraction tips for matrix-assisted laser desorption/ionization, nanoelectrospray, and LC/MS sample pretreatment in proteomics. Anal. Chem..

[bib61] Rhee H.W., Zou P., Udeshi N.D., Martell J.D., Mootha V.K., Carr S.A., Ting A.Y. (2013). Proteomic mapping of mitochondria in living cells via spatially restricted enzymatic tagging. Science.

[bib62] Richman T.R., Ermer J.A., Davies S.M.K., Perks K.L., Viola H.M., Shearwood A.M.J., Hool L.C., Rackham O., Filipovska A. (2015). Mutation in MRPS34 compromises protein synthesis and causes mitochondrial dysfunction. PLoS Genet..

[bib63] Richter R., Rorbach J., Pajak A., Smith P.M., Wessels H.J., Huynen M.A., Smeitink J.A., Lightowlers R.N., Chrzanowska-Lightowlers Z.M. (2010). A functional peptidyl-tRNA hydrolase, ICT1, has been recruited into the human mitochondrial ribosome. EMBO J..

[bib64] Richter-Dennerlein R., Oeljeklaus S., Lorenzi I., Ronsör C., Bareth B., Schendzielorz A.B., Wang C., Warscheid B., Rehling P., Dennerlein S. (2016). Mitochondrial Protein Synthesis Adapts to Influx of Nuclear-Encoded Protein. Cell.

[bib65] Ritchie M.E., Phipson B., Wu D., Hu Y., Law C.W., Shi W., Smyth G.K. (2015). limma powers differential expression analyses for RNA-sequencing and microarray studies. Nucleic Acids Res..

[bib66] Rorbach J., Gammage P.A., Minczuk M. (2012). C7orf30 is necessary for biogenesis of the large subunit of the mitochondrial ribosome. Nucleic Acids Res..

[bib67] Rorbach J., Boesch P., Gammage P.A., Nicholls T.J.J., Pearce S.F., Patel D., Hauser A., Perocchi F., Minczuk M. (2014). MRM2 and MRM3 are involved in biogenesis of the large subunit of the mitochondrial ribosome. Mol. Biol. Cell.

[bib68] Rozanska A., Richter-Dennerlein R., Rorbach J., Gao F., Lewis R.J., Chrzanowska-Lightowlers Z.M., Lightowlers R.N. (2017). The human RNA-binding protein RBFA promotes the maturation of the mitochondrial ribosome. Biochem. J..

[bib69] Ruzzenente B., Metodiev M.D., Wredenberg A., Bratic A., Park C.B., Cámara Y., Milenkovic D., Zickermann V., Wibom R., Hultenby K. (2012). LRPPRC is necessary for polyadenylation and coordination of translation of mitochondrial mRNAs. EMBO J..

[bib70] Shetty D.K., Kalamkar K.P., Inamdar M.S. (2018). OCIAD1 Controls Electron Transport Chain Complex I Activity to Regulate Energy Metabolism in Human Pluripotent Stem Cells. Stem Cell Reports.

[bib71] Sievers F., Wilm A., Dineen D., Gibson T.J., Karplus K., Li W., Lopez R., McWilliam H., Remmert M., Söding J. (2011). Fast, scalable generation of high-quality protein multiple sequence alignments using Clustal Omega. Mol. Syst. Biol..

[bib72] Sonnhammer E.L.L., Von Heijne G., Krogh A. (1998). A hidden Markov model for predicting transmembrane helices in protein sequences. Proc. Int. Conf. Intell. Syst. Mol. Biol..

[bib73] Spremulli L., Kraus B.L. (1987). Bovine mitochondrial ribosomes: effect of cations and heterologous dissociation factors on subunit interactions. Biochem. Biophys. Res. Commun..

[bib74] Suzuki T., Suzuki T. (2014). A complete landscape of post-transcriptional modifications in mammalian mitochondrial tRNAs. Nucleic Acids Res..

[bib75] Suzuki T., Terasaki M., Takemoto-Hori C., Hanada T., Ueda T., Wada A., Watanabe K. (2001). Structural compensation for the deficit of rRNA with proteins in the mammalian mitochondrial ribosome. Systematic analysis of protein components of the large ribosomal subunit from mammalian mitochondria. J. Biol. Chem..

[bib76] Suzuki T., Terasaki M., Takemoto-Hori C., Hanada T., Ueda T., Wada A., Watanabe K. (2001). Proteomic analysis of the mammalian mitochondrial ribosome. Identification of protein components in the 28 S small subunit. J. Biol. Chem..

[bib77] Suzuki T., Nagao A., Suzuki T. (2011). Human mitochondrial tRNAs: biogenesis, function, structural aspects, and diseases. Annu. Rev. Genet..

[bib78] Tu Y.T., Barrientos A. (2015). The Human Mitochondrial DEAD-Box Protein DDX28 Resides in RNA Granules and Functions in Mitoribosome Assembly. Cell Rep..

[bib79] Wanschers B.F.J., Szklarczyk R., Pajak A., van den Brand M.A.M., Gloerich J., Rodenburg R.J.T., Lightowlers R.N., Nijtmans L.G., Huynen M.A. (2012). C7orf30 specifically associates with the large subunit of the mitochondrial ribosome and is involved in translation. Nucleic Acids Res..

[bib80] Westermann B. (2010). Mitochondrial fusion and fission in cell life and death. Nat. Rev. Mol. Cell Biol..

[bib81] Wredenberg A., Lagouge M., Bratic A., Metodiev M.D., Spåhr H., Mourier A., Freyer C., Ruzzenente B., Tain L., Grönke S. (2013). MTERF3 regulates mitochondrial ribosome biogenesis in invertebrates and mammals. PLoS Genet..

[bib82] Zaganelli S., Rebelo-Guiomar P., Maundrell K., Rozanska A., Pierredon S., Powell C.A., Jourdain A.A., Hulo N., Lightowlers R.N., Chrzanowska-Lightowlers Z.M. (2017). The pseudouridine synthase RPUSD4 is an essential component of mitochondrial RNA granules. J. Biol. Chem..

