# Supplementary material for: MitoRibo-Tag Mice Provide a Tool for In Vivo Studies of Mitoribosome Composition
Source: Cell Rep. 2019 Nov 5;29(6):1728–1738.e9. doi: 10.1016/j.celrep.2019.09.080 (PMC6859486; doi:10.1016/j.celrep.2019.09.080)
Supplement: Document S1. Figures S1–S6 and Table S3 [file mmc1.pdf]

Cell Reports, Volume *29*

Supplemental Information

MitoRibo-Tag Mice Provide a Tool for *In Vivo* Studies of Mitoribosome Composition

Jakob D. Busch, Miriam Cipullo, Ilian Atanassov, Ana Bratic, Eduardo Silva Ramos, Thomas Schöndorf, Xinping Li, Sarah F. Pearce, Dusanka Milenkovic, Joanna Rorbach, and Nils-Göran Larsson

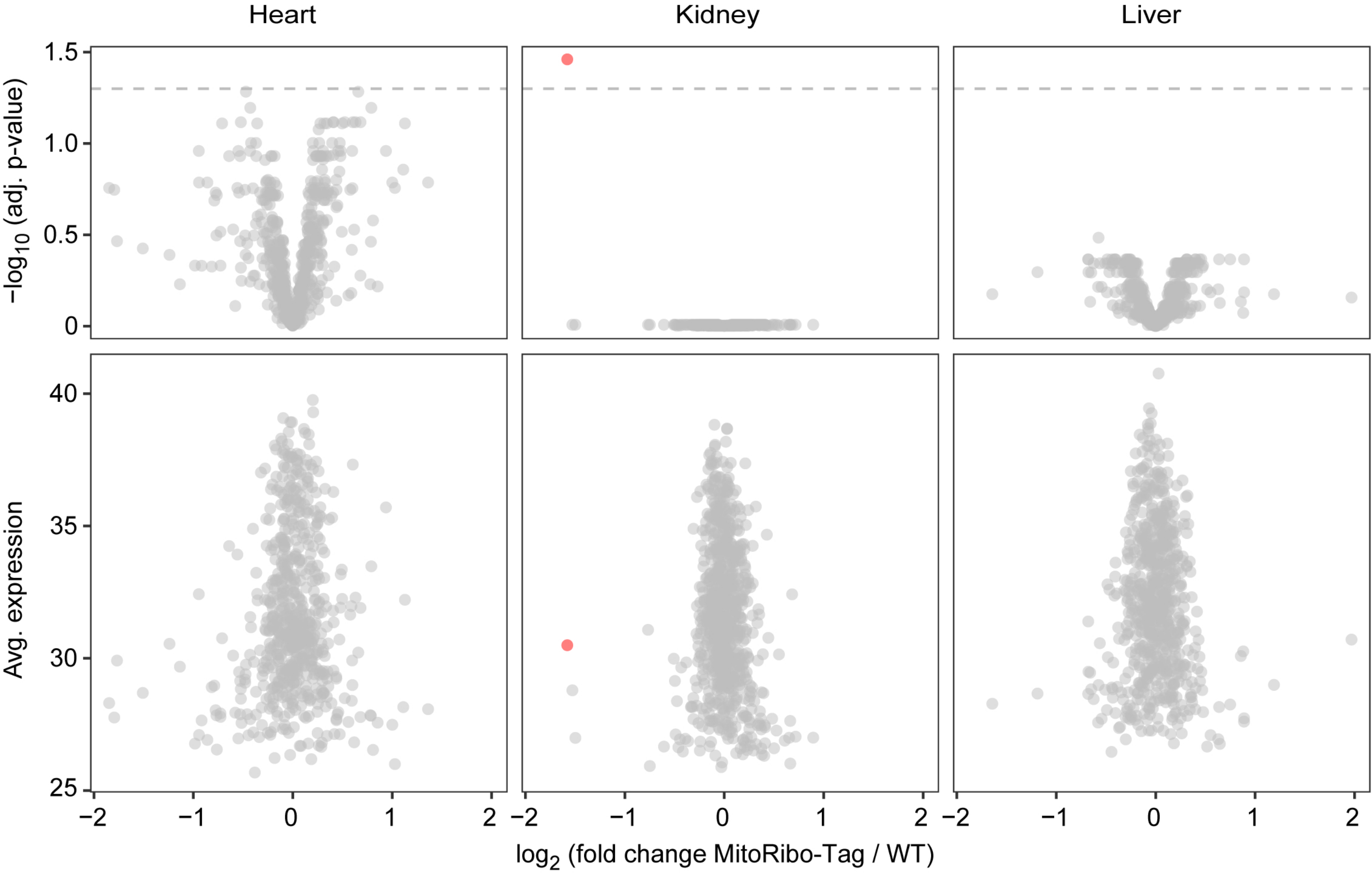

Heart
Kidney
Liver
-log10 (adj. p-value)
1.5
1.0
0.5
0
Avg. expression
40
35
30
25
−2
−1
0
1
2
log2 (fold change MitoRibo-Tag / WT)

Figure S1

Figure S1 – Mass spectrometry of protein steady-state levels in mitochondria of MitoRibo-Tag mice (related to Figure 1)

Comparison of protein steady-state levels in heart, liver and kidney mitochondria of MitoRibo-Tag (*T/T*) versus wild-type (+/+, WT) mice as determined by LFQ-MS/MS. The x-axis represents the fold change, and the y-axis indicates the adjusted p-value (upper panel) or the average expression value (lower panel) of MitoRibo-Tag vs. WT. The dashed line represents a 5 % false discovery rate. The red dot in the panels of kidney mitochondria represents the mitochondrial carbamoyl phosphate synthase. The results represent five biological replicates per genotype and tissue. See also supplemental data Tables S1 and S2.

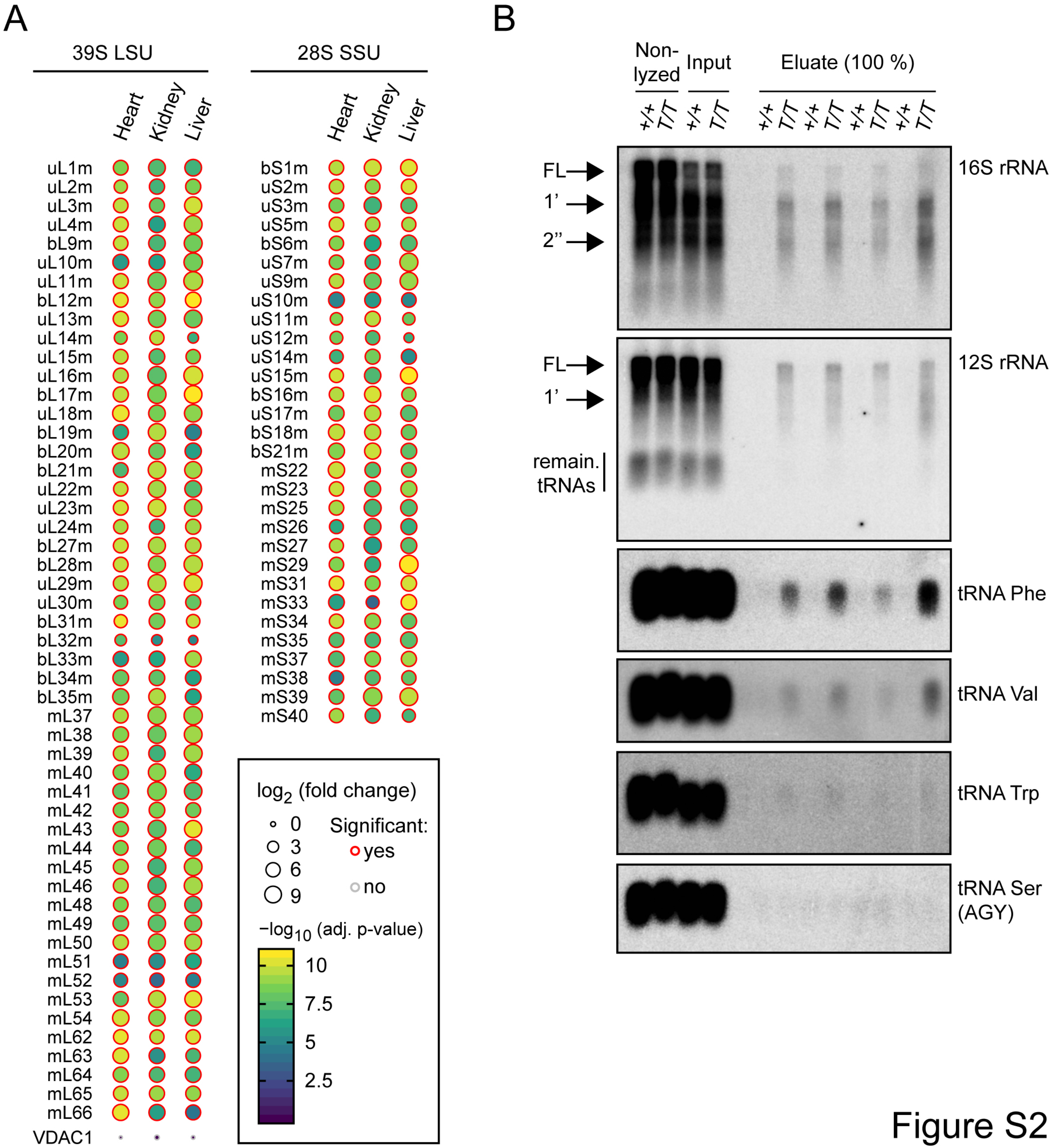
A
39S LSU
28S SSU
Heart
Kidney
Liver
uL1m
uL2m
uL3m
uL4m
bL9m
uL10m
uL11m
bL12m
uL13m
uL14m
uL15m
uL16m
bL17m
uL18m
bL19m
bL20m
bL21m
uL22m
uL23m
uL24m
bL27m
bL28m
uL29m
uL30m
bL31m
bL32m
bL33m
bL34m
bL35m
mL37
mL38
mL39
mL40
mL41
mL42
mL43
mL44
mL45
mL46
mL48
mL49
mL50
mL51
mL52
mL53
mL54
mL62
mL63
mL64
mL65
mL66
VDAC1
bS1m
uS2m
uS3m
uS5m
bS6m
uS7m
uS9m
uS10m
uS11m
uS12m
uS14m
uS15m
bS16m
uS17m
bS18m
bS21m
mS22
mS23
mS25
mS26
mS27
mS29
mS31
mS33
mS34
mS35
mS37
mS38
mS39
mS40
log2 (fold change)
0
3
6
9
Significant:
yes
no
−log10 (adj. p-value)
10
7.5
5
2.5
B
Non-lyzed
Input
Eluate (100 %)
+/+
T/T
FL
1'
2''
16S rRNA
12S rRNA
remain. tRNAs
tRNA Phe
tRNA Val
tRNA Trp
tRNA Ser (AGY)

Figure S2

Figure S2 – Overview of mitoribosome components purified from heart, liver and kidney mitochondria of MitoRibo-Tag mice (related to Figure 3)

(A) LFQ-MS/MS of mL62-FLAG co-immunoprecipitated mitoribosomal proteins from liver, kidney and heart mitochondria of MitoRibo-Tag (*T/T*) versus wild-type (+/+, WT) mice. The lysis buffer was supplemented with digitonin. The $\log_2$ (fold change; dot size), $-\log_{10}$ (adjusted p-value; blue to yellow color) as well as significant enrichment (red circle) are highlighted. Proteins with an adjusted p-value of less than 0.05 (5 % false discovery rate) were termed significant. The depicted result is representative of five biological replicates per genotype. See also supplemental data Tables S1 and S2. (B) Representative northern blot of mitochondrial rRNAs and tRNAs co-purified with mitoribosomes from liver mitochondria of MitoRibo-Tag (*T/T*) or WT mice. Non-lyzed and lyzed samples correspond to one µg total mitochondrial RNA prior the co-immunoprecipitations. Full-length (FL), one prime and two prime indicate full length or cleaved 12S and 16S rRNAs, respectively.

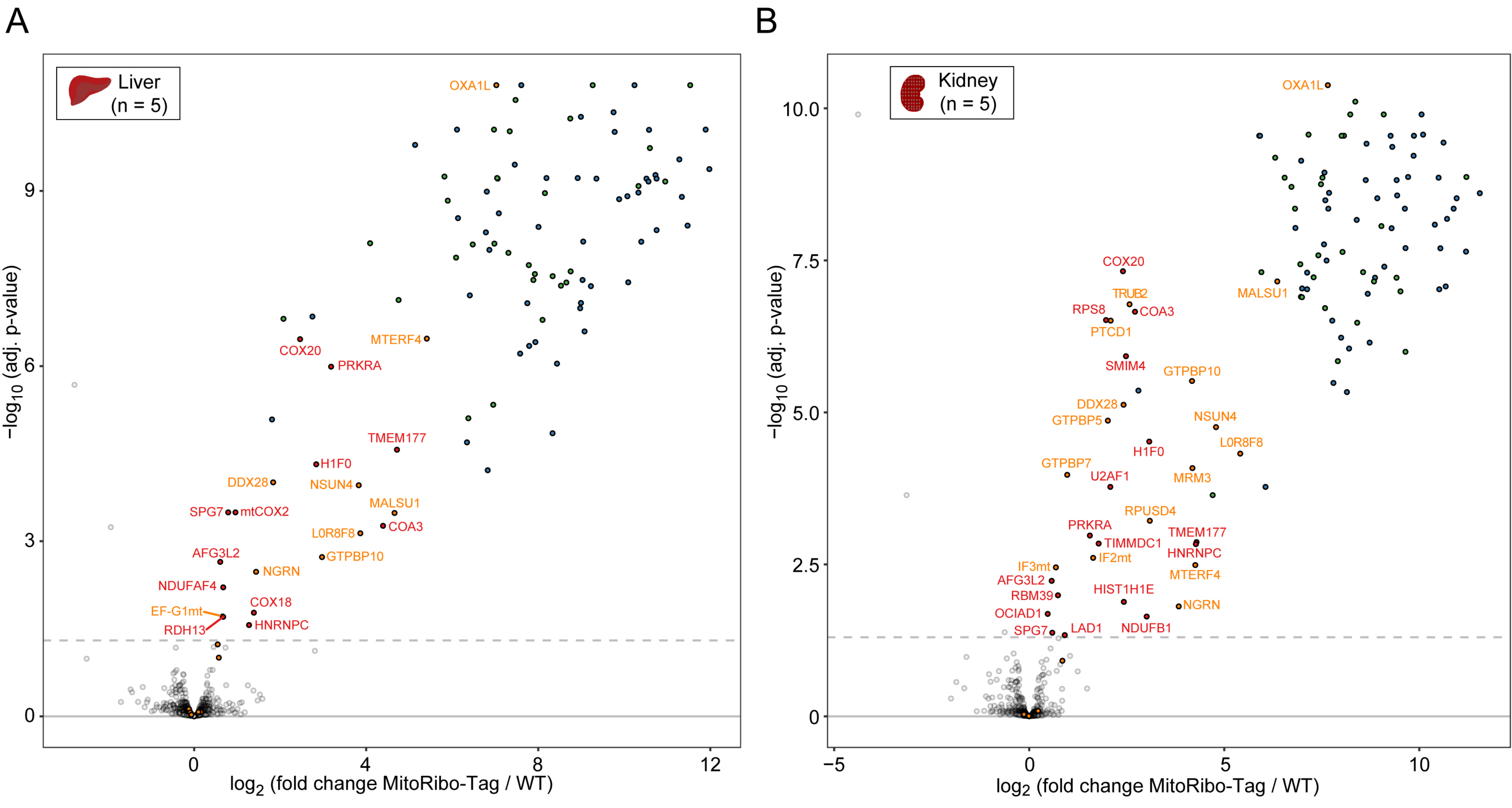
A
Liver
(n = 5)
OXA1L
MTERF4
COX20
PRKRA
TMEM177
H1F0
DDX28
NSUN4
MALSU1
SPG7
mtCOX2
COA3
L0R8F8
GTPBP10
AFG3L2
NGRN
NDUFAF4
COX18
EF-G1mt
RDH13
HNRNPC
−log10 (adj. p-value)
log2 (fold change MitoRibo-Tag / WT)
B
Kidney
(n = 5)
OXA1L
COX20
TRUB2
MALSU1
RPS8
COA3
PTCD1
SMIM4
GTPBP10
DDX28
GTPBP5
NSUN4
L0R8F8
H1F0
GTPBP7
U2AF1
MRM3
RPUSD4
PRKRA
TMEM177
TIMMDC1
HNRNPC
IF2mt
IF3mt
MTERF4
AFG3L2
HIST1H1E
RBM39
NGRN
OCIAD1
SPG7
LAD1
NDUFB1
−log10 (adj. p-value)
log2 (fold change MitoRibo-Tag / WT)

Figure S3

Figure S3 – The mitoribosome-interactomes from liver and kidney mitochondria of MitoRibo-Tag mice (related to Figure 3)

LFQ-MS/MS analysis of the mitoribosome-interactome in liver (A) and kidney (B) mitochondria of MitoRibo-Tag (*T/T*) versus wild-type (+/+, WT) mice. The lysis buffer was supplemented with digitonin. Mitoribosomal proteins are colored in green (SSU) and blue (LSU). Translation-associated proteins are highlighted in orange and other significantly enriched proteins in red. The x-axis represents the fold change and the y-axis the adjusted p-value. The dashed line represents a 5 % false discovery rate. The results are representative for five biological replicates per genotype and tissue. See also supplemental data Tables S1 and S2.

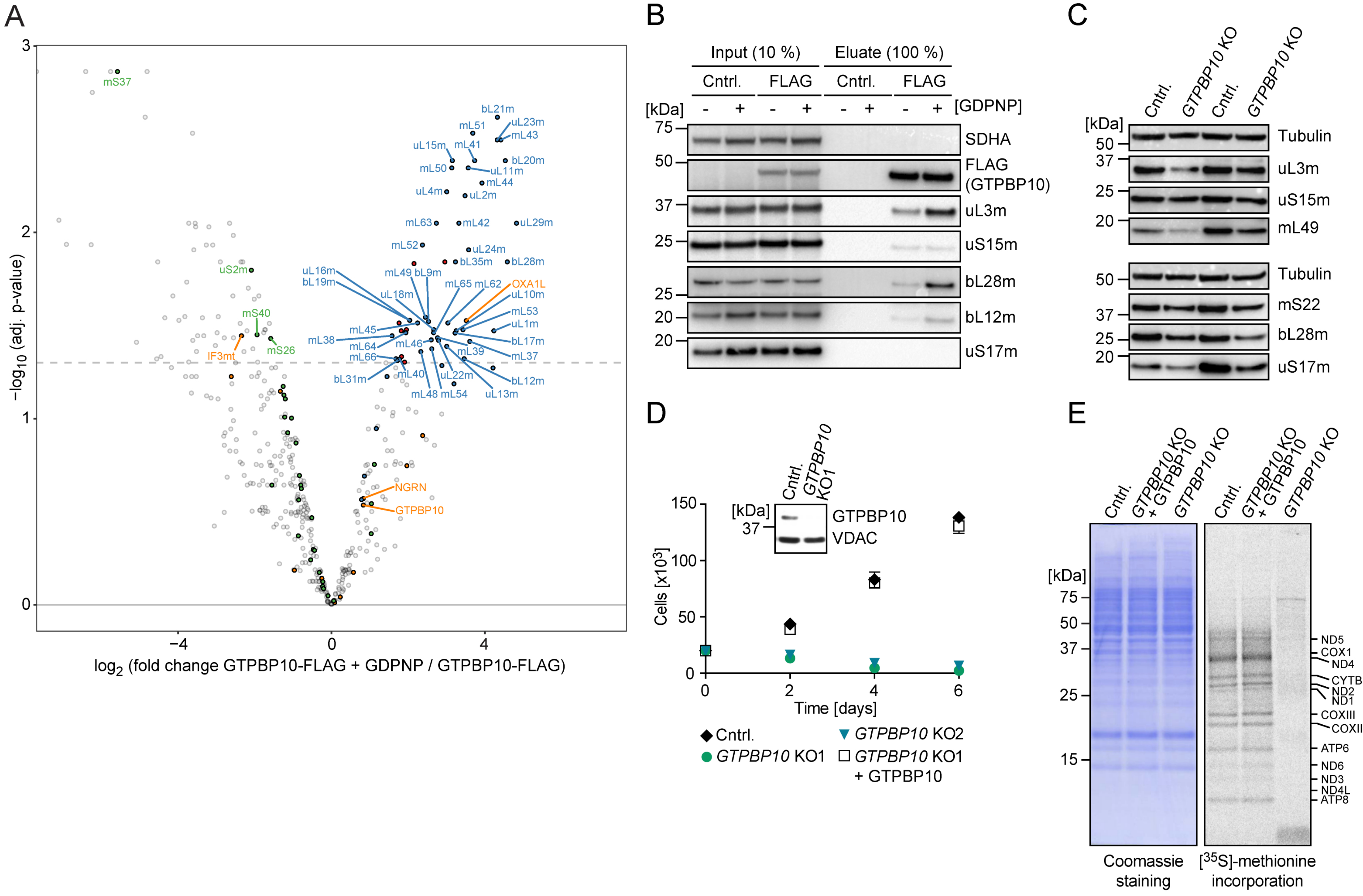

A
B
Input (10 %)
Eluate (100 %)
Cntrl.
FLAG
[kDa]
[GDPNP]
SDHA
FLAG (GTPBP10)
uL3m
uS15m
bL28m
bL12m
uS17m
C
Cntrl.
GTPBP10 KO
Tubulin
uL3m
uS15m
mL49
mS22
bL28m
uS17m
D
GTPBP10
VDAC
Cells [x10^3]
Time [days]
Cntrl.
GTPBP10 KO1
GTPBP10 KO2
GTPBP10 KO1 + GTPBP10
E
GTPBP10 KO + GTPBP10
Coomassie staining
[^{35}S]-methionine incorporation
ND5
COX1
ND4
CYTB
ND2
ND1
COXIII
COXII
ATP6
ND6
ND3
ND4L
ATP8
−log$_{10}$ (adj. p-value)
log$_2$ (fold change GTPBP10-FLAG + GDPNP / GTPBP10-FLAG)

Figure S4

Figure S4 – GTPBP10 interacts with the mitoribosome in a GTP-dependent manner (related to Figure 4)

(A) LFQ-MS/MS of GTPBP10-FLAG-associated complexes in mitochondria of HEK293T cells in the absence or presence of GDPNP (n = 3). The lysis buffer was supplemented with triton X-100. Mitoribosomal proteins are colored in green (SSU) and blue (LSU). Translation-associated proteins are highlighted in orange and other significantly enriched proteins in red. The x-axis represents the fold change and the y-axis the adjusted p-value. The dashed line represents a 5 % false discovery rate. See also supplemental data Tables S1 and S2. (B) Representative western blots of GTPBP10-FLAG co-immunoprecipitation complexes in the presence (+) or absence (-) of non-hydrolyzable GDPNP. The lysis buffer was supplemented with triton X-100. (C) Western blot analysis of protein steady-state levels in cell lysates of GTPBP10 knock-out versus HEK293T control (Cntrl.) cells (n = 2). (D) Western blot of GTPBP10 levels in relation to VDAC (loading control; n = 2) and cell growth in knock-out and control HEK293T cells (n = 3). The depicted error bars represent the standard error of the mean from three replicate experiments. (E) In cellulo [35S]-methionine pulse labeling of HEK293T cells to assess mitochondrial translation in control, GTPBP10 knock-out and GTPBP10 knock-out cells with re-expression of GTPBP10 (n = 3).

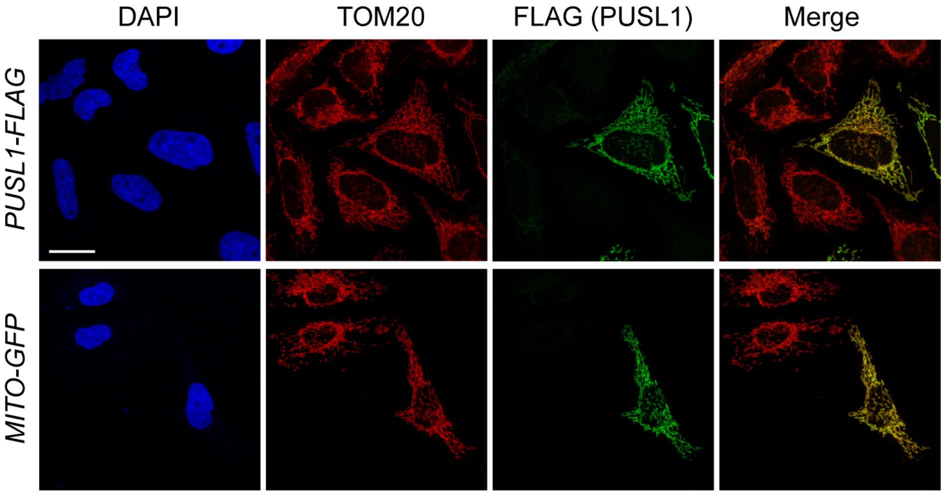

Figure S5

Figure S5 – PUSL1 localizes to mitochondria (related to Figure 5)

Representative confocal images of human HeLa cells transiently transfected with a pcDNA5/FRT/TO vector encoding *PUSL1-FLAG* or mitochondrial-targeted GFP (*Mito-GFP*). Cells were immunostained using anti-TOM20 (mitochondrial marker) and anti-FLAG antibodies. Nuclear DNA was stained with DAPI. Scale bars correspond to 20 µm. Staining patterns were observed in three biological replicates.

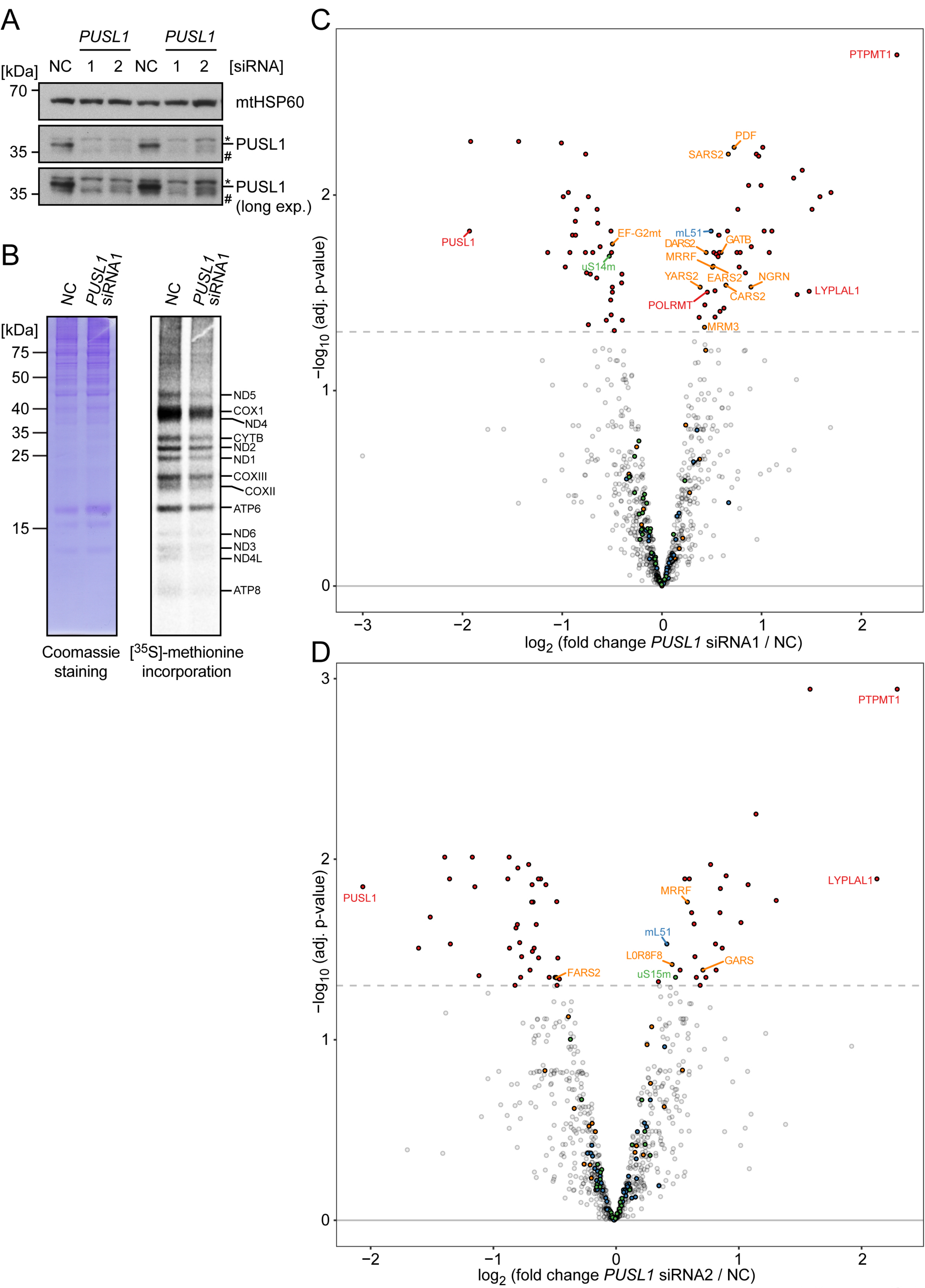
A
PUSL1
PUSL1
[kDa] NC 1 2 NC 1 2 [siRNA]
70
mtHSP60
35
* PUSL1
#
35
* PUSL1
# (long exp.)
B
NC
PUSL1 siRNA1
NC
PUSL1 siRNA1
[kDa]
75
50
40
35
25
15
ND5
COX1
ND4
CYTB
ND2
ND1
COXIII
COXII
ATP6
ND6
ND3
ND4L
ATP8
Coomassie staining
[35S]-methionine incorporation
C
PTPMT1
PDF
SARS2
PUSL1
EF-G2mt
mL51
DARS2
GATB
MRRF
uS14m
YARS2
EARS2
NGRN
LYPLAL1
POLRMT
CARS2
MRM3
−log10 (adj. p-value)
log2 (fold change PUSL1 siRNA1 / NC)
D
PTPMT1
PUSL1
LYPLAL1
MRRF
mL51
L0R8F8
GARS
uS15m
FARS2
−log10 (adj. p-value)
log2 (fold change PUSL1 siRNA2 / NC)

Figure S6

Figure S6 – siRNA-mediated knock-down of PUSL1 decreases mitochondrial de novo translation and mildly affects translation-associated proteins (related to Figure 6)

(A) Western blot analysis of PUSL1 steady-state levels in mitochondria after siRNA treatment with negative control (NC), *PUSL*1-targeting siRNA1 (1) or siRNA2 (2), showing two of three replicate experiments. Asterisk (*) and number sign (#) indicate cross-reacting bands using the commercial PUSL1 antibody. (B) Representative in cellulo [^{35}S]-methionine pulse-labeling experiment in HEK293T cells treated with negative control or *PUSL1*-siRNA1 (n = 3). (C, D) LFQ-MS/MS analysis of protein steady-state levels in mitochondria upon knock-down of *PUSL1* using the denoted siRNA1 (C) or siRNA2 (D) versus the negative control (NC) in HEK293T cells. Mitoribosome proteins are colored in green (SSU) and blue (LSU). Translation-associated proteins are highlighted in orange and other significantly enriched proteins in red. The x-axis represents the fold change and the y-axis indicates the adjusted p-value of *PUSL1* siRNA1 or siRNA2 vs. NC. The dashed line represents a 5 % false discovery rate. The results are representative for three replicates per genotype. See also supplemental data Tables S1 and S2.

CellPress

Table S3. Oligonucleotides used in this study (Related to Key Resources Table)

REAGENT or RESOURCE	SOURCE	IDENTIFIER
Oligonucleotides		
MitoRibo-Tag (*mL62-Flag* knock-in) forward primer: 5'-AGCAACAGAGGATTGTGAGTCCC-3'	This study	N/A
MitoRibo-Tag (*mL62-Flag* knock-in) reverse primer: 5'-TGGGAATGGAGCTCAATG G-3'	This study	N/A
Mterf4 loxP forward primer: 5'-CTCCCGGGTGGCGTGTG-3'	Cámara et al., 2011	N/A
Mterf4 loxP reverse primer: 5'-TTTGGGGGAAGGAATCTGTCG-3'	Cámara et al., 2011	N/A
Ckmm Cre forward primer: 5'-CACGACCAAGTGACAGCAAT-3'	This study	N/A
Ckmm Cre reverse primer: 5'-AGAGACGGAAATCCATCG	This study	N/A
CRISPR/Cas9 gRNA1_top_GTPBP10: 5'-CACCGCCAGCCACAAACCGTTTCCG-3'	This study	N/A
CRISPR/Cas9 gRNA1_bottom_GTPBP10: 5'-AAACCGGAAACGGTTTGTGGCTGGC-3'	This study	N/A
CRISPR/Cas9 gRNA2_top_GTPBP10: 5'-CACCGACGAGGATAACCCATTCCAC-3'	This study	N/A
CRISPR/Cas9 gRNA2_bottom_GTPBP10: 5'-AAACGTGGAATGGGTTATCCTCGTC-3'	This study	N/A
CRISPR/Cas9 gRNA exon1_sense_PUSL1: 5'-CACCGCACAAGATAGCGCGCGCGCA-3'	Haeussler et al., 2016	N/A
CRISPR/Cas9 gRNA exon1_antisense_PUSL1: 5'-AAACTGCGCGCGCGCTATCTTGTGC-3'	Haeussler et al., 2016	N/A
CRISPR/Cas9 gRNA exon3_sense_PUSL1: 5'- CACCGCGCCGAGCGGCTGAATTCCG-3'	Haeussler et al., 2016	N/A
CRISPR/Cas9 gRNA exon3_antisense_PUSL1: 5'-AAACCGGAATTCAGCCGCTCGGCGC-3'	Haeussler et al., 2016	N/A
